# Is Human Auditory Cortex Organization Compatible With the Monkey Model? Contrary Evidence From Ultra-High-Field Functional and Structural MRI

**DOI:** 10.1093/cercor/bhy267

**Published:** 2018-10-24

**Authors:** Julien Besle, Olivier Mougin, Rosa-María Sánchez-Panchuelo, Cornelis Lanting, Penny Gowland, Richard Bowtell, Susan Francis, Katrin Krumbholz

**Affiliations:** 1 Medical Research Council Institute of Hearing Research, School of Medicine, University of Nottingham, University Park, Nottingham, UK; 2 Department of Psychology, American University of Beirut, Riad El-Solh, Beirut, Lebanon; 3 Sir Peter Mansfield Imaging Centre, School of Physics and Astronomy, University of Nottingham, University Park, Nottingham, UK; 4 Department of Otorhinolaryngology, Radboud University Medical Center, University of Nijmegen, Nijmegen, Netherlands

**Keywords:** auditory core, fMRI, frequency selectivity, Heschl’s gyrus, myelin, tonotopy

## Abstract

It is commonly assumed that the human auditory cortex is organized similarly to that of macaque monkeys, where the primary region, or “core,” is elongated parallel to the tonotopic axis (main direction of tonotopic gradients), and subdivided across this axis into up to 3 distinct areas (A1, R, and RT), with separate, mirror-symmetric tonotopic gradients. This assumption, however, has not been tested until now. Here, we used high-resolution ultra-high-field (7 T) magnetic resonance imaging (MRI) to delineate the human core and map tonotopy in 24 individual hemispheres. In each hemisphere, we assessed tonotopic gradients using principled, quantitative analysis methods, and delineated the core using 2 independent (functional and structural) MRI criteria. Our results indicate that, contrary to macaques, the human core is elongated perpendicular rather than parallel to the main tonotopic axis, and that this axis contains no more than 2 mirror-reversed gradients within the core region. Previously suggested homologies between these gradients and areas A1 and R in macaques were not supported. Our findings suggest fundamental differences in auditory cortex organization between humans and macaques.

## Introduction

**Figure 1.**
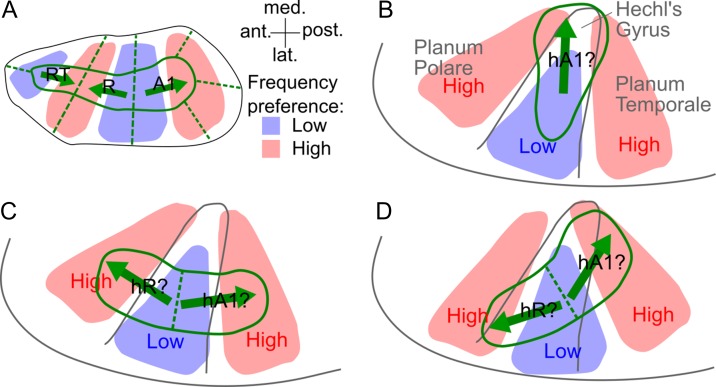
Models of monkey and human auditory cortex organization (on the left supratemporal plane). (*A*) Layout of the 3 core areas (A1, R, RT) and surrounding belt areas in the macaque auditory cortex according to Kaas and Hackett (2000). The solid green lines indicate borders between core and belt areas, and the dashed lines, borders within the core or belt. The blue and red shading shows regions responsive to lower and higher frequencies, respectively, and the green arrows show the directions of the connecting tonotopic gradients. (*B*) “Mediolateral” model of the human core based on postmortem anatomical results with presumed human homolog of macaque area A1 (hA1). (*C*) “Anteroposterior” model based on recent fMRI studies of human tonotopy with presumed human homologs of monkey core areas A1 and R (hA1, hR). (*D*) “Oblique” model intended to reconcile the mediolateral and anteroposterior models (Baumann et al. 2013).

The organization of the rhesus macaque auditory cortex is well established based on anatomical and physiological data (Kaas and Hackett 2000) and, despite a paucity of similar data from other primate species, is commonly assumed to be a good model of the organization of auditory cortex in humans. In macaques, up to 3 primary areas, A1, R, and RT, are stacked anteroposteriorly to form an elongated “core” region, and are surrounded by a “belt” of secondary areas (Fig. 1*A*; Kaas and Hackett 2000). The core and medially and laterally adjoining belt areas share a common tonotopic axis, which runs parallel to the core’s long, or anteroposterior, axis. As a result, tonotopic gradients in anteroposteriorly adjacent areas are mirror-symmetric (in both core and belt) and the borders between such areas are marked by tonotopic gradient reversals. In contrast, borders between core and medially and laterally adjoining belt areas are not marked by gradient reversals, but by changes in other properties, including frequency selectivity and myelin content (Morel et al. 1993; Kosaki et al. 1997).

Postmortem anatomical studies have suggested that the human core is also elongated, like the macaque core, but rotated to a more mediolateral direction, parallel to the long axis of Heschl’s gyrus (HG), the main macroanatomical feature on the human superior temporal plane (Rivier and Clarke 1997; Morosan et al. 2001; Wallace et al. 2002). Under the assumption that the human core and main tonotopic axis are parallel as in macaques, this would imply that human tonotopic gradients are also oriented parallel to the long axis of HG (Fig. 1*B*). This view has been supported by early neuroimaging studies of human tonotopy (Engelien et al. 2002; Formisano et al. 2003; Seifritz et al. 2006; reviewed in Saenz and Langers 2014), but also by some recent studies that have assessed tonotopic gradients within a predefined “core” region, based on an independent functional or structural criterion (Dick et al. 2012; Moerel et al. 2012; Schönwiesner et al. 2015). Such studies, however, have disagreed on the number of tonotopic gradients contained within the core region, with some finding only one (Dick et al. 2012; De Martino et al. 2015) or, at most, 2 gradients (Schönwiesner et al. 2015), but others finding 3 (Moerel et al. 2012), as in macaques. The medial-most gradient, pointing towards the medial end of HG, has been interpreted as the human homolog of macaque area A1 (referred to as hA1; Fig. 1*B*), and any more lateral gradients, as homologs of R and RT (hR and hRT; Moerel et al. 2014).

A dramatically different view has been suggested by several other recent neuroimaging studies that have assessed tonotopic gradients across the entire auditory-responsive region. These studies have consistently found 2 tonotopic gradients on HG, running approximately perpendicular, rather than parallel, to the gyrus’s long axis (Talavage et al. 2004; Humphries et al. 2010; Da Costa et al. 2011; Striem-Amit et al. 2011; Langers 2014). According to the monkey model, this finding would imply that the human core is elongated anteroposteriorly (Fig. 1*C*; Woods et al. 2009; Humphries et al. 2010), rather than mediolaterally as suggested by the postmortem results. The more posterior of the 2 gradients has been interpreted as hA1, and the more anterior as hR (reviewed in Saenz and Langers 2014). In an attempt to reconcile these 2 conflicting views, Baumann et al. (2013) recently suggested an “oblique” configuration, whereby the human tonotopic axis and core are both oriented at a slight angle to HG (Fig. 1*D*).

Here, we set out to test these different models of human auditory cortex organization by measuring the orientations of the core and tonotopic gradients in 24 individual human hemispheres using high-resolution, ultra-high-field (7 T) magnetic resonance imaging (MRI). The core was delineated based on independent functional and structural MRI criteria, and the tonotopic gradients were assessed using quantitative and unbiased analysis methods. Our results confirm the postmortem anatomical finding that the human core is elongated mediolaterally, parallel to the long axis of HG, but also suggest that the predominant tonotopic axis in humans, both across the entire auditory-responsive region and within the core, is oriented anteroposteriorly, approximately perpendicular to HG’s long axis and thus perpendicular to the core. Our results are inconsistent with all 3 previous models of human auditory cortex organization (Fig. 1), and challenge their common underlying assumption that human auditory cortex organization is similar to that in macaques.

## Material and Methods

### General Outline

This study involved 12 normal-hearing subjects with no history of neurological or otological disease (4 males; age range: 30.2 ± 7.2). All gave prior informed written consent, and the study protocol was approved by the Ethics Committee of the University of Nottingham School of Medicine.

In each subject, we acquired blood-oxygen-level-dependent (BOLD) functional images in response to narrowband noise (NBN) stimuli centered at 7 different frequencies ranging from 251 to 6009 Hz. In addition, we also acquired 2 structural data sets to estimate local cortical myelin content. One was weighted for longitudinal relaxation time, T1, and the other for macromolecular magnetization transfer (MT). All scanning was performed on a Philips Achieva 7 T system with a birdcage transmit, and 32-channel receive coil (Nova Medical, Wilmington, MA). Functional and structural data were acquired either in a single session (7 subjects) or in separate sessions, conducted on different days (5 subjects).

Following preprocessing, the functional data were used to construct voxelwise frequency response functions, or “tuning curves,” from which we estimated local preferred frequencies and tuning widths. The preferred frequencies were used to calculate local tonotopic gradient directions (directions of greatest change in local preferred frequency) and locate low- and high-frequency gradient reversals. The tuning widths and the better of the structural data sets (T1-weighted) were used to create 2 independent delineations of the likely region of the core.

All these analyses were performed separately within each subject and hemisphere. Group-average functional and structural maps were created after nonrigid spherical normalization based on local cortical surface morphology (Fischl et al. 1999).

### Data Acquisition

#### Functional Data Acquisition

**Figure 2.**
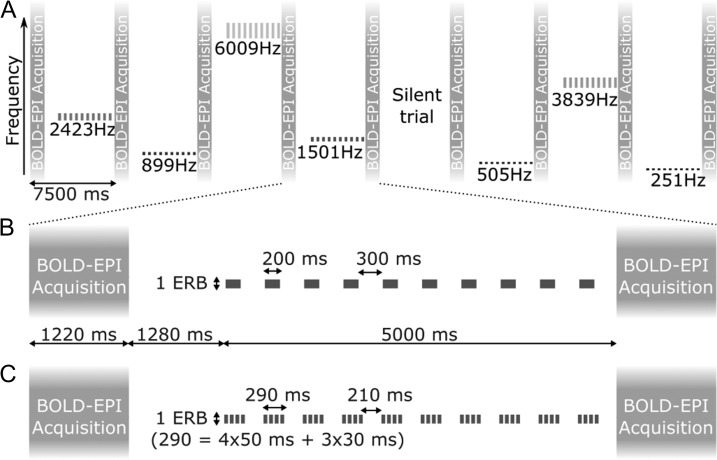
Stimulus presentation and fMRI protocol. (*A*) Timelime of 8 example fMRI trials showing all 7 stimulus frequencies and one silent trial. The long vertical bars represent the image acquisitions. The short bars show the stimulus sequences presented in between, with center frequency and bandwidth indicated on the vertical axis (representing a linear frequency scale). (*B* and *C*) Detailed timeline of a single stimulus trial presented to the first (*B*) and second (*C*) halves of the subjects.

Functional volumes were acquired with a sparse (TR = 7.5 s), T2*-weighted multi-slice, single-shot gradient echo (GE), echo-planar imaging (EPI) sequence (TE = 25 ms, SENSE factor = 3, FA = 90°). Each volume consisted of 20 contiguous axial slices, which were oriented parallel to the Sylvian fissure (1.5 mm isotropic resolution, field-of-view [FOV] = 174 × 157.68 mm^2^ [AP × RL], AP phase encoding direction) and combined with a perpendicular anterior outer-volume suppression slab. Shim coil currents were set to minimize static *B*_0_ inhomogeneities within a cuboidal region around the expected location of auditory cortex (calculated to second order; see Sanchez-Panchuelo et al. 2010, for details). Each volume took 1.22 s to acquire, leaving a 6.28-s silent gap between successive acquisitions. The experimental stimuli were presented during the last 5 s of this gap (Fig. 2*A*).

The stimuli used for the present study were presented alongside other stimuli as part of an fMR-adaptation experiment. They consisted of regular trains of NBNs with a constant center frequency and bandwidth (Fig. 2*A*). The center frequencies were chosen from 7 values between 251 and 6009 Hz (251, 505, 899, 1501, 2423, 3839, and 6009 Hz), spaced evenly on a cochlear frequency scale (based on behavioral measurements of cochlear frequency resolution; Glasberg and Moore 1990), and the bandwidths corresponded to the normal cochlear filter bandwidth at the respective center frequency (defined as equivalent rectangular bandwidth, or ERB_N_; Glasberg and Moore 1990). Across different subjects, the noises were presented either in single 200-ms bursts (first half of subjects; Fig. 2*B*), or in short sequences of four 50-ms bursts, separated by 30-ms silent gaps (second half of subjects; Fig. 2*C*). Both the single bursts and burst sequences were presented once every 500 ms. Each individual burst was gated on and off with 10-ms quarter-cosine ramps and presented at a sound pressure level (SPL) of 70 dB. To minimize differences in stimulus sensation level (level above detection threshold) across stimulus frequencies and participants, the stimuli were presented in a continuous background of equally exciting noise (filtered to contain equal energy within all cochlear filters; Glasberg and Moore 2000), set to a level of 35 dB SPL per ERB_N_. All stimuli were generated digitally at a sampling rate of 24.414 kHz using Matlab (The MathWorks, Natick, MA, USA) and digital-to-analog converted at a 24-bit amplitude resolution using a TDT System 3 consisting of an RP2.1 real-time processor and an HB7 headphone amplifier (Tucker Davis Technologies, Alachua, FL, USA). They were presented diotically through electrostatic insert earphones (EarPlug, NordicNeuroLab, Bergen, Norway) fitted with Earlink 3A foam eartips (3M Auditory systems, Indianapolis, IN, USA), after having been equalized to compensate for the low-pass characteristics of the inserts.

The functional measurements were separated into 8-min runs. Each run contained 4 trials of each of the 7 stimulus frequencies, as well as 12 silent trials (where no sound was presented other than the equally exciting noise background), presented in a random order. Most subjects completed 6 runs (yielding 6×4 = 24 trials per stimulus frequency), except subjects 3 and 6, who completed 8 and 4 runs, respectively. To remain alert, subjects watched a self-chosen silent subtitled movie.

#### Structural Data Acquisition

We measured local cortical myelin content using both a T1-weighted phase-sensitive inversion recovery (PSIR) sequence (Hou et al. 2005) and the MT ratio (Henkelman et al. 2001). The PSIR sequence was developed to provide high isotropic resolution (0.6 mm) across the whole cortex within a reasonable acquisition time (Mougin et al. 2016). A similar sequence has recently been validated in postmortem human auditory cortex (Wallace et al. 2016). In the MTR sequence, both the saturation and readout components were optimized to have high sensitivity to MT, whilst, at the same time, being robust to inhomogeneities in *B*_0_ and *B*_1_ (longitudinal and RF field strengths; Mougin et al. 2010).

##### Phase-sensitive inversion recovery

The PSIR sequence acquired 2 turbo field echo (TFE) readout trains [90 phase encodings, linear k-space encoding scheme, FA = 8°, TR = 15 ms, TE = 6.1 ms, shot-to-shot interval = 5000 ms, 0.6 mm isotropic resolution, FOV = 200 × 180.95 × 139.8 mm^3^ (AP × RL × FH) covering the whole head, acquisition time = 12 min] after each inversion pulse. The first readout train was acquired at an inversion delay (TI) of 742 ms, approximating the null point between the gray- and white-matter inversion recovery (i.e., where gray and white matter have equal but opposite signals) to maximize T1 weighting. The second train was acquired at TI = 2685 ms, when all tissues have recovered past their null points and their signals are thus predominantly weighted by proton density (PD). The PD-weighted volume was used, first, to recover the sign of the magnetization of the T1-weighted volume (Gowland and Leach 1991), and then, to remove any effects of *B*_1_ inhomogeneity from the resulting sign-corrected volume by voxelwise division (because the T1- and PD-weighted volumes are affected identically by B1 inhomogeneities; Van de Moortele et al. 2009).

##### Magnetization transfer ratio

The MTR was computed from an MT-TFE sequence consisting of 2 TFE readouts (200 phase encoding steps, low–high k-space encoding scheme, FA = 8°, TR = 12 ms, TE = 5.5 ms, 0.7 mm isotropic resolution, FOV = 192 × 173.33 × 39.90 mm^3^ covering the Sylvian fissure, acquisition time = just under 10 min), one of which was acquired after a train of off-resonance saturation pulses, and the other without saturation. The saturation pulses excite (saturate) macromolecule-bound protons (mainly myelin), and part of this saturation then transfers to the free-water protons, decreasing their available longitudinal magnetization (*M*_SAT_) compared with the unsaturated case (*M*_0_; Henkelman et al. 2001). The MTR, which reflects the amount of magnetization transfer, was computed voxelwise as MTR = (*M*_0_−*M*_SAT_)/*M*_0_, after linearly aligning *M*_SAT_ with *M*_0_ (using FLIRT in FSL v4.1.6 http://fsl.fmrib.ox.ac.uk; Smith et al. 2004). Saturation was performed with a train of 20 off-resonance (1000 Hz upfield) Gaussian-windowed, sinc-shaped RF pulses with amplitude *B*_1_ = 3.8 μT, pulse duration *D *= 20 ms, and pulse-onset interval *T* = 40 ms. The saturation train was followed by a 33-mT/m spoiler gradient of 14.6 ms duration to remove any residual transverse magnetization.

#### Auxiliary Data Acquisition

To help spatially align the functional with the structural data (see Data Analysis), we acquired 2 additional structural volumes, one T2*-weighted (3D FLASH sequence, TE = 9.9 ms, TR = 370 ms, FA = 32°, 0.5 × 0.5 × 1.5 mm^3^ resolution), and the other T1-weighted (3D MPRAGE sequence, TE = 1.79 ms, TR = 14 ms, FA = 10°, TI = 915 ms, 2 averages, 0.86 × 0.86 × 1.5 mm^3^ resolution), both with the same slice prescription as the functional volumes (henceforth referred to as “in-plane T2*- and T1-weighted volumes”).

To correct the functional images for geometric distortions due to dynamic *B*_0_ inhomogeneities (arising as a result of motion and respiration), we also acquired a static *B*_0_ field map (after shimming) using the same GE-EPI sequence as for the functional volumes, but with 2 different echo times, TE = 25 and 28 ms. This was combined with the phase information from each functional volume to estimate a dynamic *B*_0_ field map (Lamberton et al. 2007; Hahn et al. 2009), which was used to correct the corresponding magnitude image (Kadah and Hu 1997).

### Data Analysis

#### Initial Processing of Functional Data

The initial processing of the functional data was performed in the original volumetric space using mrTools (http://www.cns.nyu.edu/heegerlab, last accessed 4 October 2018). First, we motion-corrected the (distortion-corrected) functional volumes by aligning them with the volume acquired closest in time to the in-plane T2*-weighted volume using robust multiresolution alignment (Nestares and Heeger 2000). The motion-corrected time series were high-pass filtered (0.01 Hz cut-off), converted to percent-signal change (relative to the time series average), and concatenated across runs. Finally, they were fitted voxelwise with a general linear model (GLM) containing one regressor for each of the 7 stimulus frequencies (indicator function as to whether a given frequency had been presented in a given trial). The regression parameters, *β_i_*(*i* = 1–7), were used to construct the voxel tuning curves.

#### Projection Onto Cortical Surfaces and Flat Patches

Further analyses of the functional data, and all analyses of the structural data, were conducted after projection onto flattened cortical patches. Using Freesurfer v5.3.0 (http://surfer.nmr.mgh.harvard.edu/, last accessed 4 October 2018; Dale et al. 1999), we reconstructed inner and outer cortical surfaces (corresponding to the gray/white matter border and pial surface, respectively) from each subject’s high-resolution whole-head PSIR volume (Lüsebrink et al. 2013). These were then interpolated to create additional surfaces at 9 equidistant intermediate cortical depth fractions, yielding a total of 11 equidistant surfaces for each subject and hemisphere (labeled 0, 0.1, 0.2 … 1, where 0 is the inner, and 1 the outer surface). The middle surfaces (cortical depth = 0.5) were used to create flattened representations of the cortical patches surrounding the left and right supratemporal auditory cortices using the mrFlatMesh algorithm (Vista software, http://white.stanford.edu/software/, last accessed 4 October 2018). The target resolution of the flat patches was set such that each of their pixels measured approximately 0.33 × 0.33 mm^2^ on the original surfaces. The actual resolution varies across subjects and hemispheres due to flattening-related distortions.

**Figure 4.**
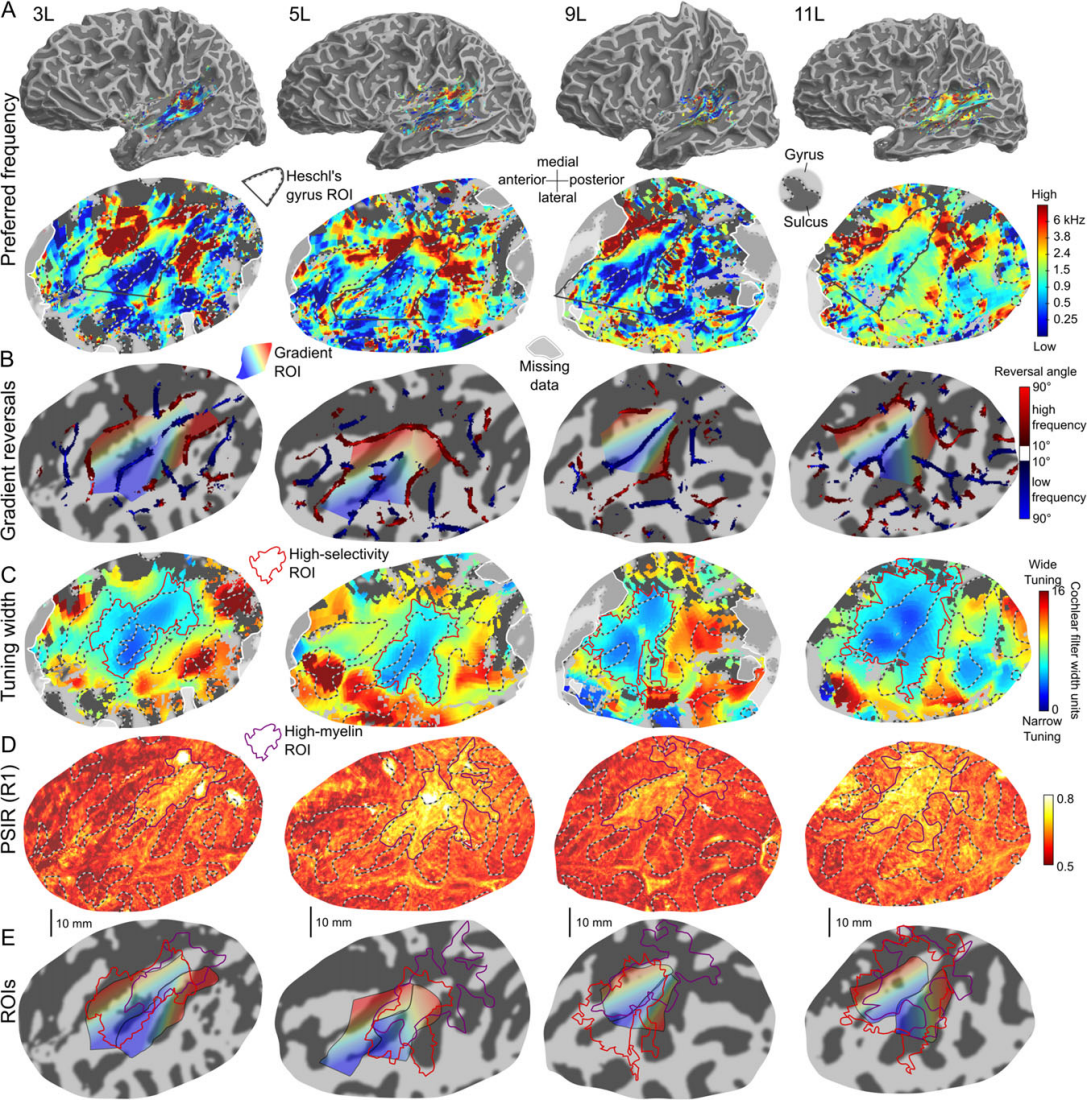
Individual functional and structural maps for 4 left example hemispheres (all 24 hemispheres are shown in Fig. S2). (*A*) Preferred-frequency maps on semi-inflated (top row) and flattened (bottom row) cortical surface reconstructions of the respective hemisphere and auditory cortex. The flat maps are plotted in the same way as in Figure 3. The black outlines show the HG ROIs (see text). White-shaded areas show missing data outside the functional acquisition stack. (*B*) Tonotopic gradient reversals derived from the individual preferred-frequency maps in panel *A*, plotted like the group-average reversals in Figure 3*C*. The rainbow-colored areas show the 2 gradient ROIs defined by the low-frequency reversal on HG and the 2 high-frequency reversals anterior and posterior to it (the color gradient shows the average gradient direction across each ROI). (*C*) Individual tuning width maps, plotted as in Figure 3*D*. The red outlines show the high-selectivity ROIs, derived by thresholding each map at an individualized criterion (see text). (*D*) Individual myelin content maps based on PSIR, plotted as in Figure 3*E*. The purple outlines show the corresponding ROIs (high-myelin ROIs) as in panel *C*. (*E*) Gradient ROIs from panel *B* overlaid with the high-selectivity (red) and high-myelin (purple) ROIs from panels *C* and *D*.

In order to project the volumetric data onto the cortical surfaces and flat patches, the MTR and functional volumes were first aligned with the PSIR volume. The MTR volume was aligned using rigid (linear) alignment. For the functional volumes, the alignment was performed in 3 steps: first, we aligned the functional volume acquired closest in time to the in-plane T2*-weighted volume with that volume using nonrigid (nonlinear) alignment (to account for any residual distortions in the functional volume); then, we aligned the in-plane T2*-weighted volume rigidly with the in-plane T1-weighted volume (treating normalized intensities as phase values in the complex plane to deal with differences in contrast), and, finally, we aligned the in-plane T1-weighted volume rigidly with the PSIR volume. All rigid alignments were performed using robust multiresolution alignment in mrTools (Nestares and Heeger 2000) and the nonrigid alignment was performed using FNIRT in FSL. The projection of the volumetric data onto the 11 cortical surfaces and flat patches was performed by nearest-neighbor interpolation. Due to the relatively small depth of the functional acquisition stack, it was impossible to image the entire supratemporal plane in all hemispheres. In Figures 4 and S2, any missing data are indicated by semitransparent white shading.

#### Processing of Structural (PSIR, MTR) Data

Like the raw PSIR data, MTR data are affected by *B*_1_ inhomogeneity. The effect can be minimized by regressing out the *B*_1_ error (spatial variation in *B*_1_ from its nominal value; Ropele et al. 2005). Given that we measured *B*_1_ error in only half of the subjects, we used the PD-weighted PSIR volume instead as a proxy. On average, the PD-weighted PSIR data explained 15.8% of the MTR variance across pixels of the flat cortical patch. Direct *B*_1_ error measurements in half of the subjects (using the Variable Flip Angle method; Yarnykh 2007) yielded a similar average proportion of explained variance (*R*^2^ = 19.7% vs. 16.7%, *P* = 0.11), as well as qualitatively similar corrected MTR maps.

MRI estimates of cortical myelin content have been found to correlate with cortical thickness (Glasser and Van Essen 2011) and cortical curvature (Sereno et al. 2013). Part of this correlation may arise because the relationship between cortical layers and cortical depths varies with these parameters (Waehnert et al. 2014). To minimize this possible bias, we regressed cortical curvature (output of Freesurfer) and thickness (distance between inner and outer cortical surfaces) out of the PSIR and MTR data (after *B*_1_ correction) at each cortical depth. Group-average depth-wise *R*^2^ values ranged between 2.1% and 8.3% for the PSIR data (at cortical depths of 1 and 0.7, respectively), and between 1.3% and 9.7% for the MTR data (at cortical depths of 0.1 and 1).

To create group-average and individual PSIR and MTR maps, the PSIR and MTR data were averaged across the middle 5 cortical depths (0.3–0.7) and then minimally smoothed along the cortical surface using an isotropic 2D Gaussian kernel with a full width at half-maximum (FWHM) of ~2 mm (6 pixels).

#### Further Analyses of Functional Data

##### Estimation of preferred frequencies and frequency tuning widths

Previous studies have estimated local preferred frequencies and frequency tuning widths by fitting the voxel tuning curves with bell-shaped functions (e.g., Gaussians) and assessing the fitted functions’ modes and widths (Moerel et al. 2012), or by calculating the tuning curves’ centroids

(1) $$C=\sum_{i}\left(\beta_{i}\cdot F_{i}\right)/\sum_{i}\beta_{i}$$

and spreads

(2) $$S=\sqrt{\sum_{i}{\left(F_{i}-C\right)}^{2}/\sum_{i}\beta_{i}}$$

where *F_i_* are the stimulus frequencies and *β_i_* are the corresponding response sizes (Schönwiesner et al. 2015). Each method has advantages and disadvantages, and so, to achieve optimal results, we here used elements of both methods where they appeared best suited.

Fitting is susceptible to noise and here required spatial smoothing to achieve robust results. Therefore, we opted not to use fitting to estimate the preferred frequencies, in order not to obscure any of their potentially small-scale spatial variations. Instead, we first calculated the centroids and spreads of the measured voxel tuning curves (unsmoothed) as described in Eqs. 1 and 2 (with *F_i_* expressed in cochlear frequency units, and *β*_i_ thresholded at zero to eliminate negative weights). The centroids, which have previously been taken unmodified as preferred-frequency estimates (Humphries et al. 2010; Schönwiesner et al. 2015), are strongly biased towards the middle of the stimulus frequency range (values at the range edges can only occur if the responses to all other frequencies are zero). Here, we endeavored to correct this bias. We constructed a set of hypothetical Gaussian tuning curves (defined on a cochlear frequency scale, like the measured curves), with preferred frequencies (modes) spanning the entire audible range (0–16.2 kHz), and tuning widths (standard deviations) ranging from 0.4 to 20 normal cochlear filter bandwidths (ERB_N_; at 1 kHz, 0.4 and 20 ERB_N_ correspond to 0.04 and 2.19 octaves, respectively). We sampled the hypothetical curves at the 7 stimulus frequencies and calculated their centroids and spreads in the same way as for the measured curves. Each measured curve was then paired with the hypothetical curve that best matched its calculated centroid and spread (in terms of minimum sum of squared differences), and the mode of the best-matching hypothetical curve was taken as the measured curve’s unbiased preferred-frequency estimate. To create group-average and individual preferred-frequency maps, we projected the unbiased preferred-frequency estimates onto the flat cortical patches and averaged them across the middle 5 cortical depths. Excluding data from superficial depths has been shown to improve the interindividual consistency of tonotopic maps (Ahveninen et al. 2016). No further smoothing was applied to retain a maximum of spatial detail.

The reliability of the debiased preferred-frequency maps was tested using a split-half test, that is, by running the debiasing procedure on 2 separate halves of each individual’s dataset and computing the correlation between the resulting estimates. Across the 24 hemispheres, the split-half Pearson correlation coefficient for the debiased preferred-frequency maps ranged between 0.09 and 0.50 (mean = 0.31). For comparison, the correlation coefficient for the original biased preferred-frequency maps ranged between 0.14 and 0.52 (mean = 0.35). A direct comparison between the biased and unbiased preferred-frequency maps is presented in Supplementary Figure S1. This figure shows that, apart from increasing the range of estimated preferred frequencies, the debiasing procedure did not qualitatively alter the tonotopic patterns. The increase in the range of preferred-frequency estimates was driven by voxels whose responses increased or decreased monotonically as a function of stimulus frequency and were thus best matched by Gaussian tuning curves with modes outside the stimulus frequency range.

Calculating the centroids and spreads of voxel tuning curves (Eqs 1 and 2 ) requires eliminating negative response sizes (here, by thresholding the *β_i_* at zero) and this may bias the spreads by changing the tuning curve shapes. Therefore, we here opted not use the spreads for estimating the tuning widths, but to use fitting instead. The fitting was performed with Gaussian functions using nonlinear least-squares minimization (trust-region-reflective; *lsqcurvefit* in Matlab). The functions were defined by 4 parameters: their mode, width, height and any negative offset in the baseline. The best-fitting functions’ widths were taken as the tuning width estimates. Before fitting, we first smoothed the voxelwise tuning curves across and along the cortical surface. For this, we projected the tuning curves onto the flat cortical patches and recentered each curve to its centroid, rounded to the nearest stimulus frequency. We then averaged the recentered tuning curves across all 11 cortical depths and smoothed them with a ~3-mm (9-pixel) isotropic 2D Gaussian kernel. The increased averaging across cortical depths (compared with that used for the preferred-frequency maps) was intended to minimize the need for smoothing along the cortical surface.

The preferred-frequency and tuning width estimates were derived only for auditory-responsive voxels, defined as voxels that responded significantly to at least one of the 7 stimulus frequencies. Statistical significance was tested with an *F*-test of all 7 sound-versus-silence contrasts. The significance level was corrected for multiple comparisons within a bilateral auditory cortex ROI (false discovery rate [FDR], using an adaptive step-up procedure (Benjamini et al. 2006)). The ROI was defined by selecting all voxels with *P* < 0.05 uncorrected at the expected location of supratemporal auditory cortex (in volumetric space) and convolving with a sphere of 7.5-mm (5-voxel) radius in order to create a connected region (excluding voxels outside the brain). A less stringent selection criterion (response size > 1% signal change for at least one stimulus frequency) was used for the figures.

##### Assessment of tonotopic gradients and gradient reversals

To assess the directions of tonotopic gradients, we submitted the estimated preferred frequencies to the 2D Sobel operator (*imgradient* in Matlab), which calculates local gradient vectors between each pixel and its immediate neighbors. Gradient directions were averaged across all cortical depths but not smoothed along the cortical surface. The local gradient vectors were used to construct the distributions of gradient directions within the core regions, to calculate group-average local gradient directions and to test the consistency of gradient directions across individuals (see Group-Average Maps).

Reversals in local gradient direction were located using the automated, unbiased procedure proposed by Schönwiesner et al. (2015). This procedure calculates the relative angles between neighboring local gradient directions by first detecting gradient sign reversals along 180 equidistant orientations between 0° and 179° and then counting the number of orientations along which a sign reversal occurred. Gradient reversals can then be identified by thresholding the relative angle maps at a criterion value (here, 10°; the exact choice of the criterion did not materially affect the locations or shapes of the detected reversals). In order to create interpretable gradient reversal maps at the individual-subject level, the local gradient vectors had to be smoothed with a 2D Gaussian kernel of ~6-mm (18-pixel) FWHM (after averaging across all 11 cortical depths, as for the gradient directions).

#### Regions of Interest

To quantify the spatial relations between tonotopic gradients and core regions at the individual level, we defined a set of 5 surface ROIs on each hemisphere’s flat cortical patch: 1 for HG, 2 for the core region (delineated by tuning width and myelin content), and 2 for 2 mirror-symmetric tonotopic gradients located on HG (see Results for details).

For all ROIs, we computed the centroids by averaging the 2D pixel coordinates within them and expressing the resulting average coordinates in a new coordinate system, defined by the centroid and long-axis orientation of the HG ROI. The long axis of the HG ROI was derived by conducting a singular value decomposition (*svd* in Matlab) of the cortical curvature gradients within the ROI and taking the second right-singular vector. The curvature gradients were derived by submitting the curvatures to the 2D Sobel operator (using *imgradientxy* in Matlab). The long axes of the core ROIs were derived by applying singular value decomposition to the ROIs’ 2D pixel coordinates and taking the first right-singular vectors.

For the tonotopic gradient ROIs, we calculated the average gradient direction across each ROI by averaging the local gradient vectors derived earlier (see Assessment of tonotopic gradients and gradient reversals), and then taking the direction of the resulting average vector. We also estimated the orientation of the low-frequency gradient reversal that separated the 2 gradients (extending along HG; see Results). To do this, we identified all clusters of contiguous low-frequency reversal pixels within the HG ROI and calculated each cluster’s long-axis orientation (by applying *svd* to their 2D pixel coordinates, as for the core ROIs above). We then estimated the overall low-frequency reversal orientation within the HG ROI as the average of the cluster orientations, weighted by the number of pixels within each cluster.

Long-axis orientations were restricted to the range between 0 and π (because they are nondirectional), whereas tonotopic gradient directions were allowed to range between −*π* and *π*. All long-axis orientations and gradient directions were expressed relative to the long-axis orientation of the respective (individual) HG ROI. Mean values and 95% confidence intervals (CIs) across hemispheres were computed using the *CircStat* (circular statistics) toolbox for Matlab (Berens 2009; nondirectional orientations were first multiplied by 2, and the resulting means and CIs then divided by 2).

Finally, we derived the overlap between each of the core and tonotopic gradient ROIs by dividing the number of pixels belonging to both ROIs by the number of pixels in the smaller of the 2 ROIs. Individual differences in the pattern of overlap for a given core ROI were characterized by the ratio of its overlap values with the 2 gradient ROIs. The overlap ratio can range from zero to infinity; a ratio of one indicates that the core ROI overlaps both gradient ROIs equally, whilst zero or infinity indicate exclusive overlap with the anterior or the posterior gradient ROI, respectively.

### Surface-Based Normalization and Group Analysis

In addition to the individual cortical surfaces, we also reconstructed cortical surfaces for the MNI single-subject template brain (http://www.bic.mni.mcgill.ca/ServicesAtlases/Colin27, last accessed 4 October 2018; Holmes et al. 1998), using the default Freesurfer analysis pipeline. We then aligned the individual left- and right-hemisphere surfaces with the corresponding MNI surfaces by aligning each surface with the default Freesurfer group template surface (fsaverage) using Freesurfer’s nonrigid spherical registration (Fischl et al. 1999). To combine data from left and right hemispheres, we also aligned each individual right-hemisphere surface with the right side of the left-right-flipped MNI surfaces (representing the left MNI hemisphere).

#### Group-Average Maps

To create group-average maps, we first resampled the left and right individual spherical surfaces to match the vertices of the left-hemisphere MNI spherical surface, and created a flattened representation of the cortical patch surrounding the left supratemporal auditory cortex of the MNI brain, as for the individual subjects. We then projected the individual maps of preferred frequency, tuning width, and myelin content (PSIR and MTR) onto the left-hemisphere MNI flat cortical patch using nearest-neighbor interpolation and averaged them pixelwise across hemispheres.

A group-average tonotopic gradient reversal map was created by applying Schönwiesner et al. (2015)’s automated reversal detection procedure to the group-average preferred-frequency map.

Group-average local tonotopic gradient directions were calculated by averaging the individual local gradient vectors pixelwise and taking the direction of the resulting group-average vectors. Before averaging, the directions of the individual gradient vectors were expressed relative to the long-axis orientation of the respective (individual) HG ROI. Gradient sign maps were created by projecting the group-average gradient vectors onto various directions, including directions parallel and perpendicular to the long axis of HG (Petkov et al. 2006; Moerel et al. 2012; Leaver and Rauschecker 2016). To test the consistency of local gradient directions across hemispheres, the individual gradient vectors were first smoothed along the cortical surface (~3-mm, or 9-pixel isotropic 2D Gaussian kernel; same as for estimating the local tuning widths) and then tested pixelwise against zero using a one-sample Hotelling *T*^2^ test, as suggested by Langers (2014). The resulting *P*-values were FDR-adjusted across the auditory-responsive voxels defined earlier (see Estimation of Preferred Frequencies and Frequency Tuning Widths). Smoothing was used to increase statistical power; without smoothing, the FDR-corrected significance level was not reached at any voxel (see Fig. S5B).

#### Comparison With a Cytoarchitectonic Atlas

The data were compared with the cytoarchitectonically defined human auditory cortical areas TE1.0, TE1.1, and TE1.2 (Morosan et al. 2001). Probability maps of these areas were generated in the MNI single-subject volumetric space using the SPM Anatomy toolbox (Eickhoff et al. 2005) and then projected onto the individual flat cortical patches (at all 11 cortical depths). For this, the left- and right-hemisphere MNI spherical surfaces were resampled to match the vertices of the corresponding individual spherical surfaces.

Based on the probability maps, we defined 5 nonoverlapping ROIs, 3 corresponding to areas TE1.0, TE1.1, and TE1.2, and the other 2 corresponding to the auditory-responsive regions anterior and posterior to these areas. At each cortical depth, a given pixel was assigned to 1 of the 3 TE1 areas if its probability of belonging to that area was both greater than 40% and greater than the probability of belonging to the other 2 areas. Spurious pixels on the parietal bank of the Sylvian fissure were excluded.

## Results

### Preferred Frequency Maps

**Figure 3.**
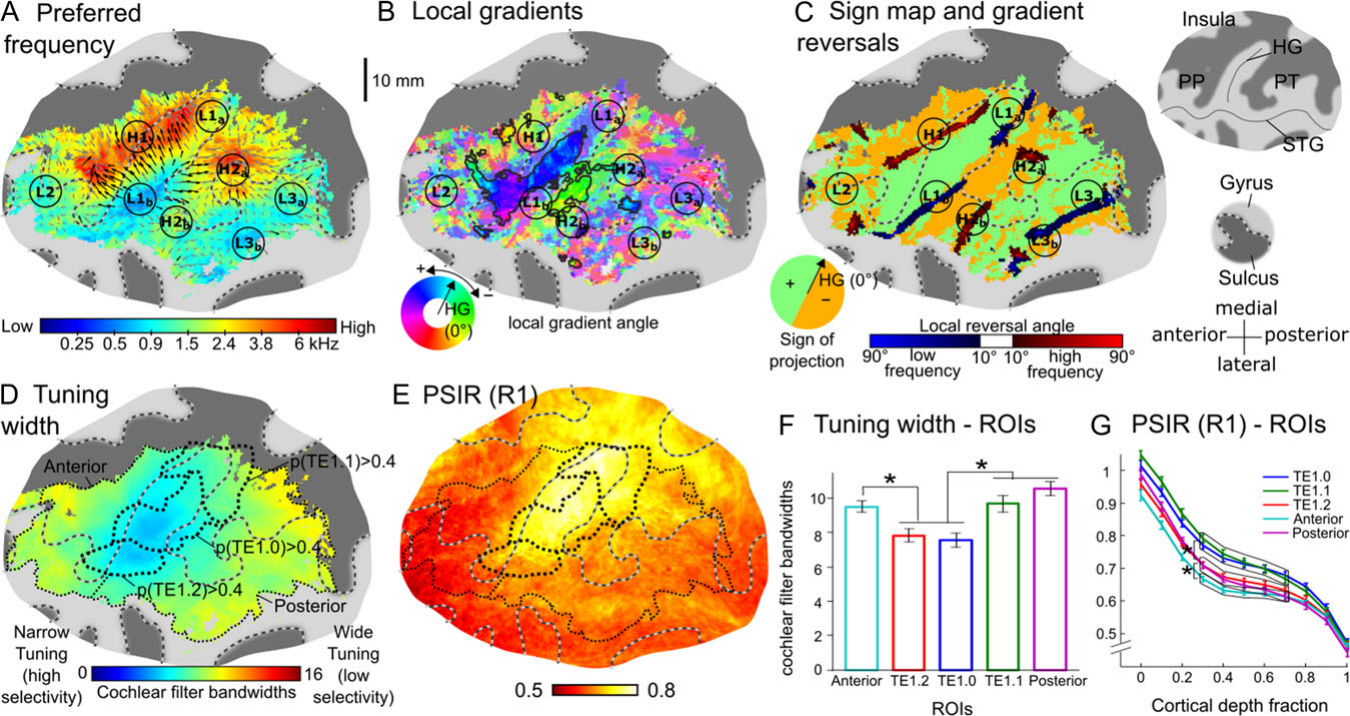
Group-average functional and structural maps from 24 left and right hemispheres (*A*–*E*) and average tuning width and myelin content within 5 cytoarchitectonically defined regions of interest (ROIs; *F*–*G*). The maps are shown on a flattened surface reconstruction of the left auditory cortex of the MNI template. (*A*) Preferred frequencies on a quasilogarithmic color scale (representing the cochlear frequency scale, see Material and Methods). Labels denote different low- (e.g., L1) or high-frequency (e.g., H1) regions. The arrows show the directions of local tonotopic gradients (black where they were significantly consistent across hemispheres, gray where they were not significant). Gyri are indicated by light-gray, and sulci by dark-gray highlight. The borders between them are shown by gray-and-black dashed lines (see insets to the right of panel *C*). (*B*) Local tonotopic gradient directions. Gradient directions are given relative to the long-axis orientation of Heschl’s gyrus (HG) in each individual hemisphere. Clusters of interindividually consistent directions (Hotelling T2, *P* < 0.05, FDR-corrected) are highlighted with black contours. (*C*) Signs of tonotopic gradients projected onto the direction perpendicular to the long axis of HG (green and orange highlight; see inset at the bottom left), and tonotopic gradient reversals derived from the group-average preferred-frequency map in panel A (darker blue and red color scales, showing reversals within regions of low or high preferred frequencies, respectively). (*D*) Local tuning widths expressed in units of cochlear filter bandwidth (see Material and Methods). The thick black dotted lines show ROIs based on the cytoarchitectonically defined areas TE1.0, TE1.1, and TE1.2 (Morosan et al. 2001), and the thin black dotted lines represent auditory-responsive ROIs anterior and posterior to these areas (referred to as “Anterior” and “Posterior” in panels *F*, *G*; note that the actual anterior and posterior ROIs were defined at the individual-hemisphere level, and thus this representation is only indicative). (*E*) Myelin content estimated with a T1-weighted phase-sensitive inversion recovery (PSIR) sequence. (*F*) Average tuning widths within cytoarchitectonically defined ROIs shown in panels *D* and *E*. (*G*) ROI-average PSIR, expressed as a function of cortical depth (0 = gray/white matter boundary, 1 = pial surface). Error bars represent the standard error of the mean across hemispheres. Stars indicate significant differences (*P* < 0.05). HG = Heschl’s gyrus; PP = planum polare; PT = planum temporale; STG = superior temporal gyrus.

Preferred frequency maps were derived from the unsmoothed voxel frequency response functions (“tuning curves”) to retain a maximum of spatial detail (see Material and Methods). Both the group-average (Fig. 3*A*) and all individual preferred-frequency maps (4 examples are shown in Fig. 4*A*, all hemispheres are shown in Fig. S2A) consistently showed a large region of low preferred frequencies on the central and lateral parts of HG (labeled L1 in Fig. 3*A*), as well as 2 large high-frequency regions adjoining this region anteromedially and posteriorly (labeled H1 and H2). In the group-average map (Fig. 3*A*), the anterior high-frequency region (H1) extended along the anterior bank of HG, reaching from the gyrus’s medial end about 3 quarters towards its lateral end, and showed very high preferred frequencies throughout its length. It was adjoined anterolaterally by a region with low to medium preferred frequencies on the planum polare (PP; labeled L2). The posterior high-frequency region (H2), on the other hand, extended along Heschl’s sulcus and onto the adjoining planum temporale (PT). It reached from the medial end of HG all the way out onto the superior temporal gyrus (STG), but showed higher preferred frequencies along its medial half (H2_a_) than along its lateral half (H2_b_). Posterolaterally, it was adjoined by a large low-frequency region (labeled L3) extending from the posteromedial edge of PT (L3_a_) anterolaterally towards STG (L3_b_). The low-frequency region on HG (L1) was wedge-shaped, growing thinner towards the gyrus’s medial end (L1_a_), and thicker towards its lateral end (L1_b_). The adjoining high-frequency regions (H1 and H2) grew commensurately closer medially, but remained separated by lower preferred frequencies at their medial end.

Whilst the individual maps (Figs 4*A* and S2A) generally supported the group-average pattern, they also showed considerable variability in detail. In many hemispheres, either or both high-frequency regions H1 and H2 were subdivided into what appeared to be distinct subregions (e.g., 3L, 5L, and 11L in Fig. 4*A*), and in some hemispheres, low-frequency region L1 appeared to be subdivided (see Table S1A for a list of typical individual preferred-frequency patterns). In the majority of hemispheres (22 of 24), H1 and H2 remained separated at their medial end by either low, or at least lower, preferred frequencies, but in 2 hemispheres (5L in Fig. 4*A* and 4R in Fig. S2A), they were fully connected through very high preferred frequencies. A split-half test indicated that the individual preferred-frequency patterns were mostly repeatable across measurements (Fig. S3), suggesting that these interindividual variations were not due to measurement noise.

### Tonotopic Gradient Directions and Gradient Reversals

Low- and high-frequency regions in preferred-frequency maps are connected by tonotopic gradients and associated with reversals, or changes, in local gradient direction. Many previous human tonotopy studies have used either qualitative or biased methods for estimating tonotopic gradient directions and locating gradient reversals. Here, we used methods that were both quantitative and unbiased. To estimate gradient directions, we calculated the local gradient vector between each pixel and its immediate neighbors within each hemisphere. We then averaged these vectors pixelwise across hemispheres to derive the group-average local gradient directions, and tested their interindividual consistency using pixelwise Hotelling *T*^2^ tests, as proposed by Langers (2014). Given that we used nonrigid spherical normalization, which aligns local surface morphological features (gyri/sulci) across individuals (Fischl et al. 1999), this would be expected to reveal gradients that exhibit a systematic relationship with the cortical surface morphology. To locate gradient reversals, we used the automated, unbiased procedure proposed by Schönwiesner et al. (2015), which detects changes in gradient sign along all possible gradient directions, rather than only a single, a priori-defined direction, such as the long-axis orientation of HG in humans (Moerel et al. 2012) or the anteroposterior axis in macaques (Petkov et al. 2006). Schönwiesner et al. (2015)’s procedure was applied to both the group-average and individual preferred-frequency maps.

The map of group-average local gradient directions (Fig. 3*B*) appeared to be divided into several mediolaterally extended strips with alternating gradient directions pointing anteriorly or posteriorly with respect to the long axis of HG (blue/purple and green/yellow hues in Fig. 3*B*). This mediolaterally alternating pattern was clearly apparent in the gradient sign map for the direction perpendicular to HG’s long axis (green and orange highlight in Fig. 3*C*), and for directions within ±45° of this direction (Fig. S4). It was further supported by the group-average gradient reversals, which extended mediolaterally along the borders between alternating gradient directions (dark red and blue shading in Fig. 3*C*). Gradient sign maps for other directions, on the other hand, showed little or no correspondence between gradient sign borders and gradient reversals (Fig. S4), highlighting the importance of using unbiased analysis methods (Schönwiesner et al. 2015). The group-average gradient reversals exhibited alternating frequency denominations (from anterior to posterior: high, low, high, low; indicated by dark red and dark blue shading in Fig. 3*C*), corresponding to successive high- and low-frequency regions in the group-average preferred-frequency map (H1, L1, H2, L3). The 3 anterior-most reversals—the low-frequency reversal corresponding to L1 on HG, and the 2 high-frequency reversals corresponding to H1 and H2 anterior and posterior to it—were also detected in all individual hemispheres (four examples are shown in Fig. 4*B*, all hemispheres are shown in Fig. S2D).

Only a small proportion of local gradient directions, located near the center of the auditory-responsive region, was significantly consistent across hemispheres (marked by black arrows in Fig. 3*A* and a black outline in Fig. 3*B*). The 2 largest clusters of significantly consistent gradient directions were located in-between the low-frequency reversal on HG (corresponding to L1) and each of the high-frequency reversals anterior and posterior to it (corresponding to H1 and H2). The more anterior of these 2 clusters (blue/purple hue in Fig. 3*B*) extended from the central crown to the lateral anterior bank of HG, and contained anteromedially pointing gradient directions within its medial two-thirds (blue hue on central HG), and anteriorly pointing gradient directions within its lateral third (blue–purple hue on the lateral anterior bank of HG). Anterolaterally, the anterior cluster was connected with a narrow strip of significantly consistent gradient directions pointing medially along the direction of HG’s long axis (light blue hue in Fig. 3*B*). Presumably, these gradient directions connected low-frequency region L2 with the anterolateral end of high-frequency region H1. The more posterior cluster occupied a restricted central portion of the posterior bank of HG and Heschl’s sulcus, and contained uniform posteriorly pointing gradient directions throughout its expanse (green highlight in Fig. 3*B*). Smaller and more isolated clusters of significantly consistent gradient directions were contained between the posterior-most high-frequency reversal in HS (corresponding to H2) and the posterior-most low-frequency reversal at the posterolateral edge of PT (corresponding to L3). Local gradient directions near the medial and lateral edges of the auditory-responsive region were oriented more mediolaterally, somewhat closer the direction of the long axis of HG, but were also noisier and less consistent across individuals.

Both the consistency testing of the group-average gradient directions and the detection of gradient reversals required some degree of spatial smoothing (see Material and Methods). Figure S5 shows that smoothing increased the interindividual consistency of local gradient directions (Fig. S5B, C), but left the average gradient directions within a given region essentially unchanged (Fig. S5A). The group-average gradient reversals remained robust down to 2-mm (for the central low-frequency reversal) or 4-mm (for all other reversals) smoothing, but became uninterpretable for lesser degrees of smoothing (see Fig. S5D). Even greater degrees of smoothing (6 mm) were required for detecting gradient reversals in individual hemispheres (data no shown).

### Core Markers

In macaques, the core is distinguished both functionally, by increased frequency selectivity (narrower frequency tuning widths), and structurally, by increased cortical myelin content (Morel et al. 1993; Kosaki et al. 1997). Previous in vivo human MRI studies have used either one or the other criterion to delineate the core (frequency selectivity: Wessinger et al. 2001; Chevillet et al. 2011; Moerel et al. 2012; Schönwiesner et al. 2015; myelin: Sigalovsky et al. 2006; Dick et al. 2012; Wasserthal et al. 2014; De Martino et al. 2015). Here, we used both criteria to create independent functional and structural in-vivo delineations of the core within the same individual hemispheres. Myelin was measured using 2 different approaches: PSIR and MT imaging (using the MT ratio, or MTR; see Material and Methods). PSIR is sensitive to the longitudinal relaxation rate, R1 (reciprocal of T1), which, like MT, depends on the local concentration of macromolecules, particularly myelin (Barkovich 2005; Bock et al. 2009).

The group-average data showed increased frequency selectivity (narrower tuning widths) and increased myelin content (PSIR and MTR) within contiguous and overlapping regions on and around HG (Fig. 3*D*,*E*, MTR map shown in Fig. S6A). The region of increased frequency selectivity (“high-selectivity region”) was mostly limited to the lateral and central parts of HG, overlapping low-frequency region L1_b_ and the anteromedially and anteriorly pointing gradient directions between L1_b_ and H1. In contrast, the region of increased myelin (“high-myelin region”) was mostly limited to HG’s central and medial parts. Both regions appeared to be elongated in the direction of the gyrus’s long axis. The PSIR measurements yielded an overall greater, and less variable, contrast than the MTR measurements, and on the PSIR map, the region of high myelination extended further into regions surrounding the medial end of HG (insula and parietal lobe). There was no significant across-voxel correlation between the voxelwise tuning width and preferred-frequency estimates when the correlation was calculated across the entire auditory-responsive region (average correlation coefficient across hemispheres: *r* = 0.015 ± 0.020; *t*-test on Fisher-transformed *r* values: *t*[23] = 0.756, *P* = 0.46), but there was a weak positive correlation when the calculation was restricted to the conjunction of the high-selectivity and high-myelin regions (*r* = 0.100 ± 0.033; *t*[23] = 3.034, *P* = 0.006).

The individual tuning width and PSIR-based myelin maps (Fig. 4*C*,*D*) supported the group-average pattern, showing regions of increased frequency selectivity and increased myelin content on and around HG that were generally elongated in the direction of the gyrus’s long axis (high-selectivity: 18 of 24 hemispheres; e.g., 3L, 5L, and 9L in Fig. 4*C*; high-myelin: 17 of 24; e.g., all hemispheres in Fig. 4*D*; see Table S1B, C and Fig. S2B, C for further examples). Some of the individual myelin maps contained additional regions of increased myelin, either located posterior to HG (7 of 24, see Table S1D and Fig. S2C for examples), consistent with previous reports (Sigalovsky et al. 2006; Dick et al. 2012), or extending posteromedially into the parietal lobe or insula (8 of 24; e.g., 5L and 9L in Fig. 4*D*, see Table S1D and Fig. S2C for further examples). In the majority of hemispheres (20 of 24, e.g., 3L, 5L, and 9L in Fig. 4*E*; see Table S1E for further examples), the high-selectivity region was mostly limited to the lateral and central parts of HG, whereas the high-myelin region was mostly limited to the gyrus’s medial and central parts, consistent with the group-average results. The individual MTR maps failed to identify any reliable patterns and were thus not analyzed further. The lesser contrast of the MTR sequence may have been due to its lower signal-to-noise ratio (compared with the PSIR sequence), or the fact that it used low-high k-space encoding (rather than linear, like PSIR), which may have blurred its tissue contrast (see Material and Methods).

### Relation Between Core Markers and Cytoarchitectonic Areas

To test how the high-selectivity and high-myelin regions compared with the expected location of the core based on postmortem anatomical data, we calculated average tuning widths and myelin contents within 3 regions of interest (ROIs) based on probabilistic maps of the cytoarchitectonically defined areas TE1.0, TE1.1, and TE1.2, which have been suggested to represent the human core (Morosan et al. 2001; thick black dotted outlines in Fig. 3*D*,*E*). For comparison, we also defined 2 noncore ROIs, comprising the auditory-responsive regions anterior and posterior to these areas (roughly corresponding to the PP and PT, respectively; thin black dotted outlines in Fig. 3*D*,*E*). Average tuning widths were narrower (indicating higher frequency selectivity) within the lateral and central cytoarchitectonic core ROIs (TE1.2 and TE1.0) compared with the medial core ROI (TE1.1) and the noncore ROIs (Fig. 3*F*; tested with a 2-way repeated-measures ANOVA; main effect of ROI: *F*[4,40] = 13.85, *P* < 10^−6^; all pairwise comparisons between TE1.2/TE1.0 and TE1.1/anterior/posterior significant at *P* < 0.003 except TE1.2 vs. TE1.1, where *P* = 0.08; *P*-values adjusted for familywise error rate). The PSIR- and MTR-based myelin measures were averaged separately at each of 11 equidistant cortical depths between the gray/white-matter border and pial surface (Figs 3*H* and S6B; see Material and Methods). Both measures decreased with increasing cortical depth, with a plateau at intermediate depths (0.3–0.7), particularly in PSIR. Within the plateau region, PSIR was significantly greater in the central and medial core ROIs (TE1.0 and TE1.1) compared with the lateral core ROI (TE1.2) and the noncore ROIs, and was lower in the anterior noncore ROI compared with all other ROIs (Fig. 3*G*; main effect of ROI: *F*[4,40] = 40.54, *P* < 10^−12^; all pairwise comparisons between TE1.1/TE1.0 and TE1.2/anterior/posterior, and between anterior and TE1.1/TE1.0/TE1.2/posterior, significant at *P* < 0.02). The MTR data showed a similar pattern across ROIs, but with greater variability (Fig. S6B).

### Quantitative Evaluation of Tonotopic Gradients and Core Regions in Individual Hemispheres

The group-average local tonotopic gradient directions (Fig. 3*B*) suggested a general anteroposterior tonotopic axis, which, as in macaques (Petkov et al. 2006), appeared to divide the auditory-responsive region into several mediolaterally extended strips of anteroposteriorly mirror-reversed gradient directions. At the same time, the regions of high-frequency selectivity and high-myelin content on HG—presumably marking the core, appeared to be elongated mediolaterally, perpendicular to the main tonotopic axis, rather than parallel to it as in macaques (Fig. 3*D*,*E*). To explore these findings further, we assessed the overlap, as well as the relative locations and orientations of the core regions and tonotopic gradients within each of the 24 individual hemispheres. All measures were derived from the unnormalized (hemisphere-specific) flat coordinate spaces to avoid excessive reliance on correspondence between cortical morphology and functional organization, which may have affected the preceding analyses involving surface-based normalization.

**Figure 5.**
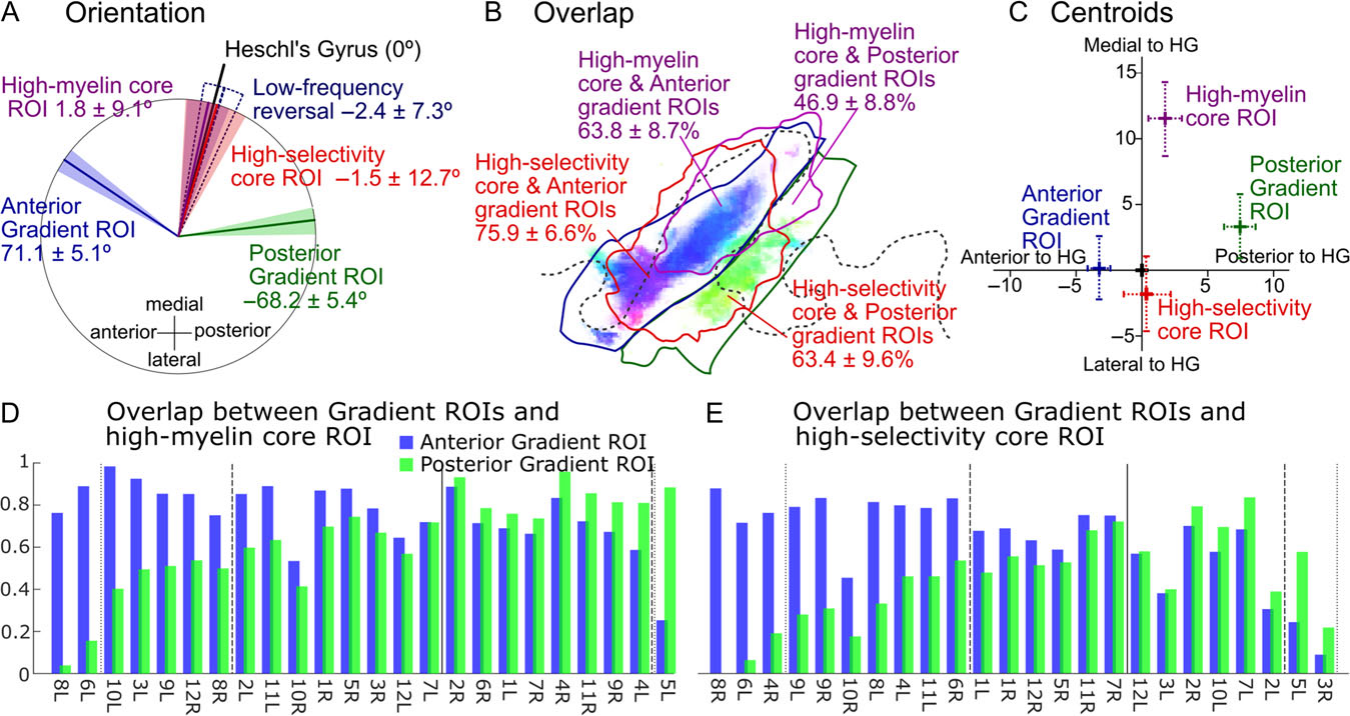
Orientations, overlap, and centroids of individual core and gradient ROIs. (*A*) Average gradient directions within the anterior and posterior gradient ROIs (blue and green), as well as long-axis orientations of the core ROIs (red and purple) and low-frequency tonotopic gradient reversal on HG (dark blue). All orientations are expressed relative to the long-axis orientation of HG (black line). The mean orientations are shown by the colored lines, and the 95% CIs by the shaded areas. (*B*) Overlap of the 2 core ROIs (high-selectivity/high-myelin) with each of the 2 gradient ROIs (anterior/posterior). The figures give the mean overlap and 95% CIs across 24 hemispheres. The ROI outlines (solid lines) are from the group-average data (Fig. 3*B*,*D*,*E*) and thus only indicative. They were derived using the same methods as for the individual ROIs (see text). The shaded highlight shows the significantly consistent local gradient directions replotted from Figure 3*B*. (*C*) Centroids of the core and gradient ROIs in relation to the centroid and long-axis orientation of the HG ROI (corresponding, respectively, to the origin and vertical axis). Crosses and error bars show the means and 95% CIs, respectively. (*D*) Degree of overlap between the high-myelin core ROI and the anterior (blue bars) and posterior (green bars) gradient ROIs in individual hemispheres. Hemispheres are sorted according to the ratio of 2 overlap values (greater overlap with the anterior gradient ROI towards the left). Vertical dotted and dashed black lines mark the points where the lesser overlap is within one-third and two-thirds of the greater overlap, respectively. The vertical solid black line indicates the point of equality between overlap values. (*E*) Same as in *D* but for the high-selectivity ROI.

Within each hemisphere, we defined 5 ROIs: one for HG (“HG ROI”), 1 for each of the 2 core markers (“high-selectivity” and “high-myelin ROIs”), and 1 for each of the 2 anteroposteriorly mirror-reversed tonotopic gradients on and just behind HG, parts of which were found to be interindividually consistent in the preceding group analysis (“anterior and posterior gradient ROIs”). The HG ROI (black outlines in Fig. 4*A*) was defined using the inflection in the cortical surface that marks the boundary between HG and the adjoining sulci. The high-selectivity and high-myelin ROIs (red and purple outlines in Fig. 4*C*,*D*) were derived by thresholding the tuning width and PSIR maps using individualized threshold criteria based on the cytoarchitectonically defined ROIs described in the previous section. The criteria were set midway between the average tuning widths/PSIR values within the TE1.0 ROI on the one hand, and the conjunction of the anterior and posterior noncore ROIs (auditory-responsive regions outside areas TE1.0, TE1.1, and TE1.2) on the other. This choice was led by the ongoing uncertainty about the primary status of areas TE1.1 and TE1.2 (Morosan et al. 2001; Moerel et al. 2014), which is further supported by the current finding that the TE1.1 ROI showed a similar average tuning width, and the TE1.2 ROI a similar average myelin content, as the noncore ROIs (Fig. 3*F*,*G*). A supplementary analysis showed that using the midpoint between the simple complements instead (i.e., the conjunction of the 3 TE1 areas vs. the auditory-responsive regions outside these areas) did not materially change the core ROIs’ locations or orientations (compare Figs 5 and S7), nor the distributions of local gradient directions contained within them (Fig. S8). The definition of the gradient ROIs (rainbow-colored regions in Figs 4*B* and S2D) was based on the tonotopic gradient reversals identified in the individual preferred-frequency maps: the anterior gradient ROI was delineated anteriorly by the high-frequency reversal anterior to HG and posteriorly by the low-frequency reversal on HG, and the posterior gradient ROI was delineated anteriorly by the low-frequency reversal on HG and posteriorly by the high-frequency reversal posterior to HG (disconnected but collinear reversals were interpreted as part of the same reversal and joined to create contiguous ROI borders).

#### Relative Orientations of Main Tonotopic Axis and Core Regions

First, we compared the long-axis orientations of the individual core ROIs (derived using singular value decomposition; see Material and Methods) with the average gradient directions within the respective anterior and posterior gradient ROIs. In addition, we also determined the orientation of the low-frequency gradient reversal separating these 2 gradients. According to the monkey model, the gradient directions should be approximately parallel to the long axes of the core ROIs, whilst the gradient reversal should extend approximately perpendicular to them. Figure 5*A* shows that, whilst the long axes of both core ROIs were essentially parallel to the long axis of HG (relative angles < 2°), the average anterior and posterior gradient directions were approximately mirror-symmetric about this axis, and the low-frequency gradient reversal separating them extended practically parallel to it—opposite to the predictions of the monkey model. On average across hemispheres, the average anterior and posterior gradient directions formed an obtuse angle of ~140° with each other, and each formed an angle of ~70° with the long axis of HG. Importantly, these angles changed little when the averaging of the gradient directions was restricted to voxels within either of the core ROIs (high-selectivity: 67.2° ± 7.3° for ant., −64.9° ± 10.8° for post.; high-myelin: 67.4° ± 5.6° for ant., −71.4° ± 8.1° for post.).

A supplementary analysis showed that these results remained largely unchanged when the anterior gradient was considered not as a single coherent gradient, but as 2 separate gradients, corresponding to the anteromedially and anteriorly pointing gradient directions in the anterior significantly consistent gradient cluster (see white outline in rightmost panel of Fig. S5B). The average gradient directions within these 2 subclusters were significantly different from each other, but pointed in a similar general direction (i.e., formed a sharp angle with each other; Fig. S9), and both were anteroposteriorly mirror-reversed to the average gradient direction within the posterior significantly consistent gradient cluster (i.e., both anterior gradient directions formed an obtuse angle with the posterior gradient direction).

#### Tonotopic Gradient Directions Contained Within the Core Regions

Next, we investigated to what degree the interindividually consistent anteromedially and posteriorly pointing gradient directions around central HG were represented within individual core regions, and whether the core regions contained any other gradient directions (e.g., near the medial end of HG) that may have failed to reach statistically significant consistency due to regional variation in surface-based registration quality. We constructed histograms of local gradient directions contained within each core ROI (high-myelin/high-selectivity) in each hemisphere, and also summed the individual histograms across hemispheres to create group-aggregate histograms.

**Figure 6.**
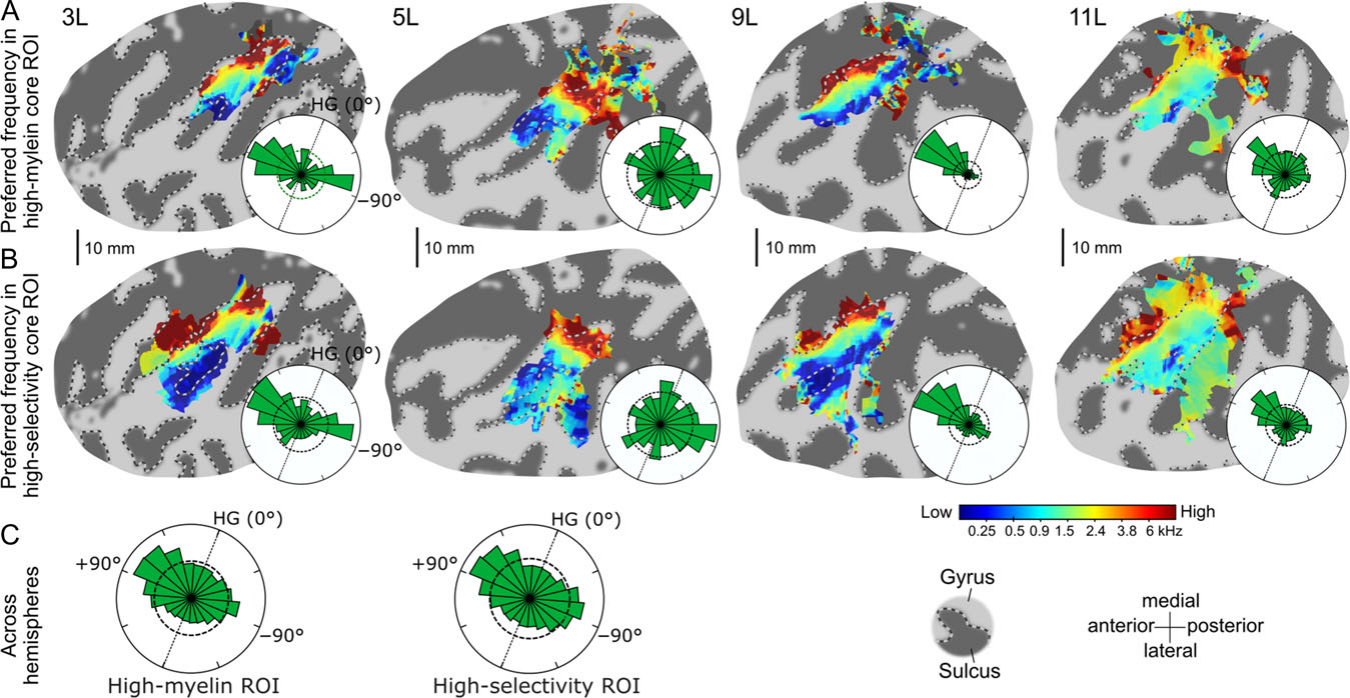
Preferred-frequency maps and histograms of local tonotopic gradient directions contained within the core ROIs. (*A*) Individual preferred frequencies (flat maps) and circular gradient direction histograms (insets) within the high-myelin ROI for the same four left hemispheres as shown in Figure 4. The histograms were normalized to fit. The straight black dotted lines within them show the long-axis orientation of HG, and the black dashed circles show circular uniform distributions representing the null hypothesis that all gradient directions were equally prevalent. (*B*) Same as in *A*, but for the high-selectivity ROI. (*C*) Aggregate gradient direction histograms across all 24 hemispheres (gradient directions from right hemisphere ROIs were sign-reversed).

Both the group-aggregate (Fig. 6*C*) and most of the individual histograms (Fig. 6*A*,*B* shows four examples; all hemispheres are shown in Fig. S8) were highly nonuniform. The group-aggregate histograms showed 2 clear modes, pointing anteromedially and posteriorly away from the long axis of HG in an approximately mirror-symmetric configuration—consistent with the significantly consistent group-average gradient directions (Fig. 3*B*) and the average gradient directions within the individual gradient ROIs (Fig. 5*A*). In both core ROIs, but particularly the high-myelin ROI, the anteromedially pointing gradient directions were more prevalent than the posteriorly pointing ones. The individual histograms (Figs 6*A*,*B* and S8) suggested a somewhat more complicated pattern, exhibiting considerable variability in both the number and direction of gradient modes. Nevertheless, the most prevalent gradient directions within individual histograms were invariably oriented close to either of the 2 anteroposteriorly mirror-reversed group-aggregate modes. In some individual histograms, more mediolaterally oriented gradient directions were also relatively prevalent (e.g., 5L in Fig. 6*A*,*B*; see Fig. S8 for further examples), but these were not sufficiently consistent across hemispheres to form discernible modes in the group-aggregate histograms.

#### Overlap Between Core and Gradient ROIs

The gradient histograms suggest that a major proportion of the individual core regions’ surface area was occupied by anteromedially and posteriorly pointing tonotopic gradient directions likely corresponding to the anterior and posterior gradient ROIs. This is supported by Figures 4*E* and S2D, which show the individual core and gradient ROIs superposed. These figures indicate that the core and gradient ROIs occupied the same general cortical region in all hemispheres, but also suggest a considerable degree of interindividual variability in the precise pattern of their overlap. To investigate this further, we quantified the extent of overlap between the core and gradient ROIs as well as the relative locations of their centroids.

On average across hemispheres, both core ROIs showed substantial overlap with both gradient ROIs, with average overlap values ranging from 47% (high-myelin with posterior gradient ROI) to as much as 76% (high-selectivity with anterior gradient ROI; Fig. 5*B*). For both core ROIs, the average overlap with the anterior gradient ROI was significantly greater than with the posterior gradient ROI (tested with paired Wilcoxon sign-rank tests; high-myelin ROI: *P* = 0.013; high-selectivity ROI: *P* = 0.049). This is consistent with the larger anteromedially pointing mode in the group-aggregate gradient direction histograms (Fig. 6*C*). A larger overlap with the anterior than posterior gradient ROI was also observed in the majority of the individual hemispheres (16/24 for high-myelin, 15/24 for high-selectivity; hemispheres to the left of the vertical solid lines in Fig. 5*D*,*E*; see example hemispheres 3L and 9L in Fig. 4*E*), although in many hemispheres, the extent of overlap was relatively similar between the 2 gradient ROIs (i.e., the lesser overlap was greater than two-thirds of the greater overlap; 12/24 for high-myelin, 16/24 for high-selectivity; hemispheres between vertical dashed lines in Fig. 5*D*,*E*; see example hemisphere 11L in Fig. 4*E*). In a few hemispheres, the core ROIs almost exclusively overlapped the anterior gradient ROI (i.e., the overlap with the posterior gradient ROI was less than one-third of that with the anterior gradient ROI; 3/24 for high-myelin, 2/24 for high-selectivity; hemispheres to the left of the vertical dotted lines in Fig. 5*D*,*E*), whereas the reverse only occurred very rarely (0/24 for high-myelin, 1/24 for high-selectivity; hemispheres to the right of the dotted lines in Fig. 5*D*,*E*; see example hemisphere 5L in Fig. 4*E*). To test whether these individual differences were related to individual differences in HG morphology, we classified individual Heschl’s gyri as either “single” or “partially” or “fully duplicated” (see Table S1F), and characterized the individual overlap patterns by taking the ratio of overlap values between each core ROI and the 2 gradient ROIs (see Material and Methods). We found no significant difference in overlap ratio between the different types of HG morphologies (tested with a Kruskal–Wallis test: high-myelin: median overlap ratio = 0.90, 0.64, and 0.37 for single, partially and fully duplicated, respectively, *P* = 0.489; high-selectivity: median overlap ratio = 0.86, 0.70, and 1.05, respectively, *P* = 0.859; note that median overlap ratios for fully duplicated HG were based on only 3 hemispheres).

The difference in the core ROIs’ average overlap with the anterior and posterior gradient ROIs was mirrored in the regions’ relative locations as quantified by their centroids. Specifically, the centroids of the anterior gradient ROI had a significantly smaller average anteroposterior distance to the centroids of the core ROIs than those of the posterior gradient ROI (Fig. 5*C*). Moreover, the anterior gradient ROI exhibited a significantly narrower average tuning width than the posterior gradient ROI (7.10 ± 0.55 vs. 7.98 ± 0.95 ERB_N_; *t*[23] = 2.09, *P* = 0.048), and also showed a marginally higher myelin content (0.72510 ± 0.017 vs. 0.711 ± 0.018; *t*[23] = 1.76, *P* = 0.091).

#### Differences Between High-Selectivity and High-Myelin Core ROIs

The group-average maps for frequency selectivity and myelin content suggested a spatial dissociation, in that frequency selectivity appeared to be elevated in the lateral and central parts of HG and myelin content in the central and medial parts. To verify this statistically, we compared the locations of the centroids of the corresponding core ROIs and measured the ROIs’ overlap with HG. This showed that the high-myelin ROI was indeed located significantly more medially along HG than the high-selectivity ROI: whilst the centroid of the high-selectivity ROI was located within 2 mm of the centroid of HG, the centroid of the high-myelin ROI was shifted by an average of 12 mm towards the gyrus’s medial end (Fig. 5*C*). In addition, the high-myelin ROI also had a lesser surface area than the high-selectivity ROI (479 ± 61 vs. 597 ± 108 mm^2^; *t*[23] = 2.51, *P* = 0.020) and showed less overlap with HG (62.8 ± 6.5% vs. 73.8 ± 4.7%; *t*[23] = 3.44, *P* = 0.022).

#### Relation Between Preferred Frequencies and Cortical Curvature

**Figure 7.**
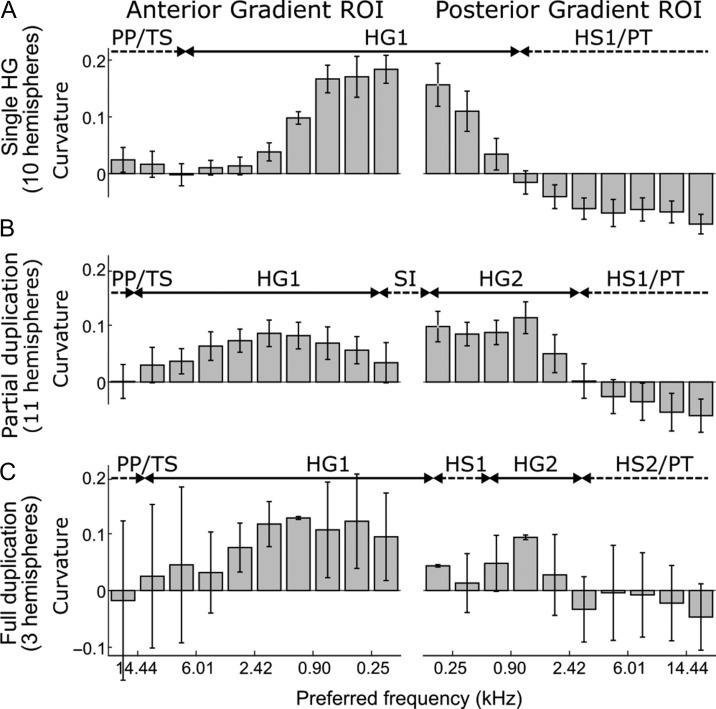
Cortical curvature as a function of local preferred frequency within the anterior and posterior gradient ROIs. Within each ROI, pixels were binned according to their preferred frequency, and cortical curvature was averaged within each bin. The results are shown separately for different HG morphologies ((*A*) single; (*B*) partially duplicated; (*C*) fully duplicated; see Table S1F). Positive curvature indicates a gyrus, and negative curvature a sulcus (see the double-arrows at the top of each panel indicating the approximate locations of relevant gyri and sulci based on the binned curvature values (labels are as in Abdul-Kareem and Sluming 2008) TS = transverse sulcus; HG1 = first Heschl’s gyrus; HS1 = first Heschl’s sulcus; SI = sulcus intermedius; HG2 = second Heschl’s gyrus; HS2 = second Heschl’s sulcus). Note that the preferred-frequency axis is reversed for the anterior gradient ROI. Error bars show the standard error of the mean across hemispheres.

Da Costa et al. (2011) suggested that the anteroposteriorly mirror-reversed tonotopic gradients on HG, which contained the significantly consistent gradient clusters in the current data, exhibit a systematic relationship between local preferred frequencies and local cortical surface morphology. To test whether a similar relationship was also evident in the current data, we plotted the relationship between the local preferred frequencies contained within each gradient ROI and the corresponding local cortical curvatures—a summary measure of cortical morphology (Fig. 7). In hemispheres with a single HG (Fig. 7*A*), the anterior gradient occupied the anterior bank (high frequencies) and crest (low frequencies) of HG, whilst the posterior gradient occupied the posterior bank of HG (low frequencies) and Heschl’s sulcus (high frequencies). A similar pattern was observed in hemispheres with a partially duplicated HG (Fig. 7*B*), and possibly also applied to hemispheres with a fully duplicated HG (Fig. 7*C*), but this is uncertain due to the low number of cases (3 of 24; see Table S1F). These results suggest that the anterior and posterior gradients were arranged asymmetrically on HG, with the anterior gradient occupying a greater proportion of the gyrus’s surface area. This was supported by the greater overlap of the HG ROI with the anterior compared with posterior gradient ROI (72.2 ± 6.3% vs. 45.5 ± 9.1%; *t*[23] = 4.58, *P* = 0.00013).

## Discussion

Previous studies have assumed that in humans, as in macaques, the auditory core is elongated parallel to the primary tonotopic axis, and subdivided perpendicular to this axis into mirror-reversed tonotopic gradients. Different studies, however, have disagreed on whether the primary tonotopic axis in humans is oriented parallel, perpendicular or oblique to the long axis of HG (reviewed in Baumann et al. 2013; Moerel et al. 2014; and Saenz and Langers 2014). To test these opposing views, we mapped tonotopy and delineated the core using a higher field strength (7 T) and a greater number of hemispheres (24) than used in previous studies. We created independent functional and structural delineations of the human core based on frequency selectivity and myelin content, and quantitatively compared the locations and orientations of the tonotopic gradients and core regions in individual, unnormalized hemispheres. Our results indicate that the organization of the human auditory cortex differs fundamentally from the monkey model, suggesting that this model will be unsuitable for guiding future in-vivo parcellation of human auditory cortex.

Though differing in detail, all 24 individual tonotopic maps showed a similar overall tonotopic pattern, with a large, wedge-shaped low-frequency region on the central and lateral parts of HG, and 2 elongated high-frequency regions extending along the gyrus’s anterior and posterior banks. A similar pattern has been found by most recent fMRI studies of human tonotopy, both in group-average and in individual data (Humphries et al. 2010; Da Costa et al. 2011; Dick et al. 2012; Langers and van Dijk 2012; Moerel et al. 2012). Consistent with previous studies that have assessed tonotopic gradients across the entire auditory-responsive region (Woods et al. 2009; Humphries et al. 2010; Da Costa et al. 2011; Striem-Amit et al. 2011; Langers and van Dijk 2012), this suggested 2 extensive tonotopic gradients pointing anteromedially and posteriorly away from the long axis of HG in the shape of a shallow “V.” The gradients formed an average angle of ~140° which was positioned approximately mirror-symmetrically about HG’s long axis, and their common low-frequency reversal extended approximately parallel to this axis. Both gradients contained large clusters of interindividually consistent local gradient directions, in line with previous results by Langers (2014). They also exhibited a systematic relationship between local preferred frequencies and local cortical surface morphology, as previously shown by Da Costa et al. (2011), and consistent with analogous relationships in other sensory modalities (Yousry et al. 1997; Benson et al. 2012). However, whilst Da Costa et al. (2011) suggested a symmetric arrangement of the 2 gradients on HG, with each gradient occupying approximately equal anterior and posterior halves of the gyrus, we here show that the arrangement is in fact asymmetric, with the anterior gradient extending posteriorly beyond the crest of HG, and the posterior gradient extending into Heschl’s sulcus and onto the adjoining PT. The group-average gradient directions suggested that the interindividually consistent gradients on HG were part of a wider anteroposterior tonotopic axis, which, as in macaques (Petkov et al. 2006), appeared to span a large proportion of the auditory-responsive region, and consist of several anteroposteriorly mirror-reversed gradients. Similar patterns of group-average gradient directions have been reported in previous studies (Langers and van Dijk 2012; Leaver and Rauschecker 2016).

The current results revealed circumscribed and overlapping regions of increased frequency selectivity and increased cortical myelin content on and around HG in all individual hemispheres, which presumably represent a correlate of the human core region. Consistent with previous postmortem (e.g., Morosan et al. 2001; Wallace et al. 2002) and in-vivo (Dick et al. 2012; Moerel et al. 2012; Wasserthal et al. 2014; De Martino et al. 2015) delineations of the human core, both regions were generally elongated in shape, and their long axes were approximately parallel to the long axis of HG (De Martino et al. 2015; Dick et al. 2017, 2012; Moerel et al. 2012; Wasserthal et al. 2014; but see Thomas et al. 2015). In line with previous reports (Dick et al. 2012; De Martino et al. 2015), we found myelin content to decrease from deeper to more superficial cortical layers, and regional myelination differences to be mostly limited to middle and deeper cortical depths. Although we found no significant correlation between frequency tuning widths and local preferred frequencies across the entire auditory-responsive region—in agreement with a previous study (Thomas et al. 2015)—a weak positive correlation emerged when we restricted the analysis to the core region. A nonzero correlation may indicate a mismatch between the quasilogarithmic cochlear frequency scale used to express the tuning width and preferred-frequency estimates on the one hand (see Material and Methods), and the cortical frequency scale on the other, and may thus suggest a change in frequency magnification between the cochlea and primary auditory cortex (Nishimura and Song 2014).

Whilst the high-selectivity and high-myelin regions showed considerable overlap, they also showed differences: the high-selectivity region generally extended roughly symmetrically from the center to the medial and lateral ends of HG, whereas the high-myelin region mainly occupied the gyrus’s medial and central parts. This difference may in part be due to residual nonlinear effects of cortical curvature and thickness (Sereno et al. 2013), which may have resulted in an overestimation of myelin content in this region. However, it is unlikely that such nonlinear effects could also explain the absence of increased myelin content in the lateral part of HG, which is independently confirmed by postmortem anatomical results (Wallace et al. 2002). Thus, the difference in lateral extent of the high-selectivity and high-myelin regions likely reflects genuine underlying functional and structural differences. It remains unclear at this point whether the true core corresponds only to the intersection of the high-selectivity and high-myelin regions on the central part of HG, or whether the differences between the high-selectivity and high-myelin regions reflect widthwise subdivisions of the core into lateral (high-frequency selectivity but low myelin content), central (both core markers high), and, possibly, medial (high-myelin content but low-frequency selectivity) areas.

The current results provide the first direct demonstration that the human core has substantial overlap with the anteromedially and, to a lesser degree, posteriorly pointing tonotopic gradients on and just behind HG, and that these gradient directions are the most prevalent within the core. Whilst there was considerable variability between individual core region’s gradient direction distributions, and although the primariness of at least part of the posteriorly pointing gradient remains uncertain, this suggests that the main tonotopic axis defined by these gradients is also the predominant tonotopic axis within the core. Thus, as in macaques (Petkov et al. 2006), the main and predominant primary tonotopic axes in humans appear to be aligned with one another. Our results also suggest that the human main and predominant primary tonotopic axes run approximately perpendicular to the long axis of HG, and thus perpendicular to the long axis of the core. In contrast, in macaques, where HG is nonexistent, the main and primary tonotopic axes run approximately parallel to the core’s long axis. Postmortem anatomical results from chimpanzees suggest that this fundamental difference may be linked to the anatomical evolution of HG (Hackett et al. 2001): in chimpanzees, the orientation of the core varies across individuals, being similar to humans (i.e., parallel HG’s long axis) in those that exhibit a HG, but similar to macaques (i.e., anteroposterior) in those that do not.

The current findings contrast with the findings of other studies that have assessed tonotopic gradients within an independently defined core region (Dick et al. 2012, 2017; Moerel et al. 2012; Schönwiesner et al. 2015), all of which have reported more mediolaterally oriented primary gradient directions, closer to the long axis of HG. This discrepancy may be due to these previous studies having used lower magnetic field strengths (3 T or 1.5 T), which would have increased sensitivity to BOLD contributions from larger draining veins located near the pial surface (Uludag et al. 2009; Uğurbil 2012; Gardumi et al. 2017) and thus decreased the inherent image resolution (by increasing the hemodynamic spatial point spread). Greater BOLD contributions from large veins at lower field strength may also have distorted the measured tonotopic patterns and increased their interindividual variability. One of the studies (Schönwiesner et al. 2015), conducted at 3 T, found individual core gradient directions ranging from almost parallel (1°), to almost perpendicular (88°) to the long axis of HG—a range twice as large as those found in the current study at 7 T (43°–88° for the anterior, and 45°–87° for the posterior gradient ROI; similar values applied when the gradient ROIs were restricted to the core regions). Whilst the previous studies assessed core gradients at both the individual and group levels, they did not statistically test the consistency of the reported gradient directions across individuals. Finally, previous delineations of the core region were generally more conservative than those derived here. For instance, Dick et al. (2012) estimated the surface area of the myelin-based core region as ~2 cm^2^, compared with ~4.8 cm^2^ in the current study. As a result, the previously delineated core regions may have included fewer of the interindividually consistent anteromedially and posteriorly pointing gradient directions on and around central HG and thus contained a greater proportion of more mediolaterally oriented gradient directions.

Apart from a general anteroposterior tonotopic axis, the current data also suggested several smaller clusters of more mediolaterally oriented gradient directions. One of these clusters, pointing posteromedially from low-frequency region L2 on PP to the anterolateral end of high-frequency region H1 anterior to HG, reached the significance threshold for interindividual consistency (Fig. 3*A*,*B*). A similar gradient has previously been suggested to represent the human homolog of the primary area RT in macaques (Moerel et al. 2014), but, contrary to this suggestion, was not included in either of the current core regions. Further, albeit nonsignificant, clusters of mediolaterally oriented gradient directions were observed near the medial edge of the auditory-responsive region. Parts of these gradients may have been included in individual core regions, particularly the high-myelin region, but may have been too weak or too variable to create a discernable mode in the core regions’ gradient direction histograms or be detected in the gradient consistency test.

Despite a reasonable overall consistency with the group-average tonotopic pattern, individual tonotopic maps also showed a high level of heterogeneity in detail. This interindividual tonotopic heterogeneity seemed to affect not only nonprimary, but also primary regions, as evidenced by the high degree of variability between individual core regions’ gradient direction histograms. Given that individual tonotopic patterns were largely repeatable across measurements, it appears unlikely that they merely represent measurement noise. Instead, they may reflect genuine idiosyncrasy in tonotopic organization. Moerel et al. (2014) suggested that some of the detailed features of individual, unsmoothed tonotopic maps may represent additional smaller tonotopic gradients that are masked by the larger anteroposteriorly oriented gradients in smoothed or group-average tonotopic maps. Alternatively, individual tonotopic features may reflect extravascular BOLD contributions from larger pial veins (Duong et al. 2003), which may follow idiosyncratic individual patterns.

It has been suggested that the anteromedially and posteriorly pointing tonotopic gradients on and just behind HG, may represent the human homologs of the primary areas A1 and R in macaques, with the posterior gradient corresponding to A1 (referred to as hA1) and the anterior gradient corresponding to R (hR; see Fig. 1*C*). This homology, however, is not supported by the current data. In macaques, A1 is more highly myelinated than R (Morel et al. 1993; Hackett et al. 2001), whereas, in the current data, the anterior gradient (supposedly hR) showed a higher average myelin content and greater overlap with the high-myelin region. In fact, the anterior gradient showed a greater overlap with both core regions than the posterior gradient (supposedly hA1). Consistent with this, a greater proportion of the core regions’ surface area was occupied by anteromedially than by posteriorly pointing local gradient directions. These results suggest that at least part of the posterior gradient does not belong to the core, but to the secondary auditory cortex, or belt (see Alternative models of human auditory cortex organization below). It is even possible that the extent of primariness of the posterior gradient varies across hemispheres: in some hemispheres, the core might encompass the posterior gradient to the same or similar degree as the anterior gradient, whereas in other hemispheres, the posterior gradient may be encompassed only partially or not at all. The latter scenario would explain the high degree of interindividual variability in the core regions’ gradient direction distributions. Either scenario would be incompatible with the idea that the posterior gradient represents the human homolog of A1.

### Alternative Models of Human Auditory Cortex Organization

Our results suggest that the human and macaque auditory cortices differ fundamentally in the way their constituent areas are laid out in relation to their tonotopy. They corroborate previous results that have suggested that, as in macaques, the main tonotopic axis in the human auditory cortex runs in an anteroposterior direction, approximately perpendicular to the long axis of HG, and they demonstrate that this general anteroposterior tonotopic axis is also the predominant tonotopic axis within the human core region. At the same time, they show that the human core is elongated in a mediolateral direction, parallel to the long axis of HG and thus approximately perpendicular to the main tonotopic axis, whereas in macaques, the core’s long axis and tonotopic axis are parallel. This refutes previous suggestions that the human core and tonotopic axis both run either perpendicular (Woods et al. 2009; Humphries et al. 2010), parallel (Formisano et al. 2003; Dick et al. 2012; Moerel et al. 2012; Schönwiesner et al. 2015), or oblique (Baumann et al. 2013) to the long axis of HG. And it implies that, in humans, the borders between primary and nonprimary areas run mostly perpendicular to the tonotopic axis (mediolaterally) and may thus be expected to be marked by tonotopic gradient reversals. In contrast, in macaques, the borders between core and belt areas run mostly parallel to the tonotopic axis (anteroposteriorly) and are thus not marked by tonotopic gradient reversals. Our results also suggest that the human core region contains at most 2 mirror-reversed tonotopic gradients along the main (anteroposterior) tonotopic axis, although, at this point, we cannot exclude the existence of additional smaller gradients with a more mediolateral orientation. Importantly, the border between these gradients ran parallel to the core region’s long axis. In contrast, in macaques, the core contains up to 3 mirror-reversed gradients and their borders run perpendicular to the core’s long axis. Finally, we show that, in humans, the anterior gradient is more highly myelinated than the posterior gradient. This contradicts the suggestion that the anterior and posterior gradients represent the human homologs of the macaque areas A1 and R, respectively. These findings call for an alternative model of human auditory cortex organization, independent of the monkey model. In this section, we interpret our results within the context of 2 different sets of postmortem anatomical results to construct 2 plausible alternatives.

**Figure 8.**
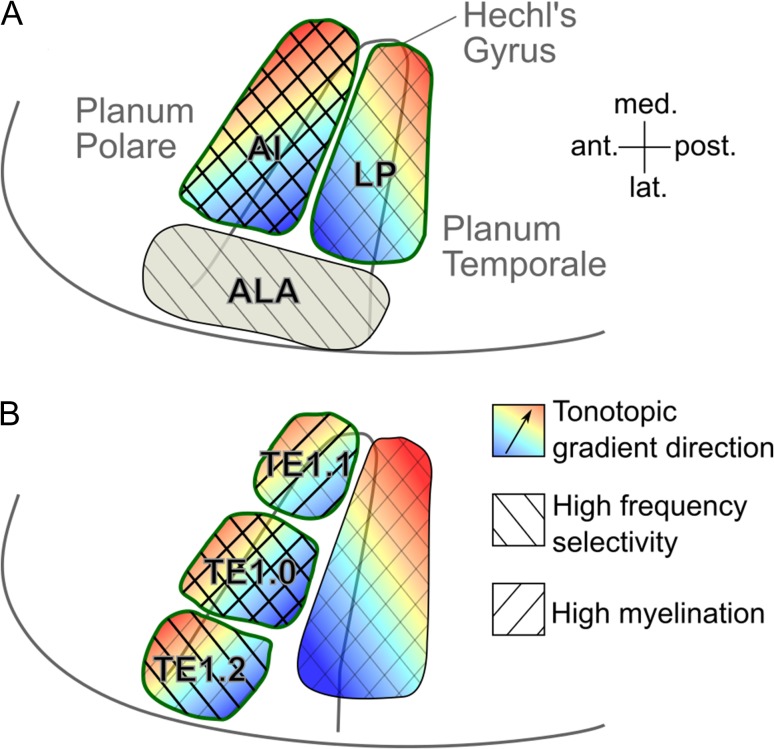
Plausible models of human auditory cortex organization based on the current data and previous postmortem anatomical results. (*A*) Likely model under the assumption that the human core contains both tonotopic gradients on HG, and only occupies the medial and central parts of the gyrus. The green outlines designate core areas. The background hatch indicates increased frequency selectivity and/or myelin content (the degree of increase is indicated by the hatch weight). The area labels are in accordance with Rivier and Clarke (1997) and Wallace et al. (2002): AI = primary auditory area; LP = lateroposterior area; ALA = anterolateral area. (*B*) Likely model under the assumption that the human core only contains the anterior tonotopic gradient on HG, and occupies the gyrus’s full length. The area labels are in accordance with Morosan et al. (2001).

The present data left open whether the human core encompasses the entire length of HG, or occupies only its central and/or medial parts, and also whether it encompasses both of the anteroposteriorly mirror-reversed tonotopic gradients on and just behind HG, or only the anterior one. If the core only occupies the central and/or medial parts of HG, our results may be compatible with the organization suggested by the postmortem studies of Rivier and Clarke (1997) and Wallace et al. (2002), both of which used a combination of histological markers, including myelin. Both studies found a primary-like area (labeled “AI”; Fig. 8*A*) showing a high degree of myelination on central and medial HG. The current data suggest that this area may correspond to part of the anteromedially pointing tonotopic gradient on anterior HG (within the region of high myelination; compare Figs 8*A* and 5*A*). In addition to AI, Wallace et al. (2002) identified a second area with primary-like properties within Heschl’s sulcus (labeled “lateroposterior area,” or LP). This area showed a lesser degree of myelination than AI and may thus correspond to the posteriorly pointing tonotopic gradient on posterior HG and in Heschl’s sulcus. Alternatively, the posterior gradient may correspond to either or both of the secondary areas PA (“posterior area”) and LA (“lateral area”), posterior and posterolateral to LP. Both postmortem studies also identified a secondary area on the lateral part of HG (labeled “anterolateral area,” or ALA), which may correspond to the lateral part of the high-selectivity region where myelination was low. It is uncertain whether this region contains 1 or 2 tonotopic gradients, and thus whether it is unitary or subdivided.

If, on the other hand, the human core encompasses the entire length of HG, our results may be compatible with the cytoarchitectonic results of Morosan et al. (2001), who identified 3 primary-like areas, TE1.0, TE1.1, and TE1.2, stacked along the length of HG (Fig. 8*B*). The 3 areas may collectively correspond to the anteromedially pointing tonotopic gradient on anterior HG, in which case the borders between them would not be marked by gradient reversals (unlike the borders between macaque areas A1, R, and RT). Area TE1.0 may correspond to the region on central HG, where the high-selectivity and high-myelin regions overlapped (compare Fig. 5*A*), and areas TE1.1 and TE1.2 may correspond to the nonoverlapping parts of the high-myelin and high-selectivity regions on medial and lateral HG, respectively. In this model, the posteriorly pointing tonotopic gradient on posterior HG and in Heschl’s sulcus would be assumed to represent a secondary area. It remains uncertain whether it represents a unitary area or is subdivided like the core.

Of course, other schemes—possibly in between these 2 models, or including additional, more mediolaterally oriented tonotopic gradients near the medial or lateral ends of HG—would also be conceivable. Independent measurements of further anatomical and functional markers will be necessary to arrive at a conclusive model of human auditory cortex organization. Previous promising approaches include cortical connectivity (Upadhyay et al. 2007; Cammoun et al. 2015; Li et al. 2015), temporal response properties (Seifritz et al. 2002), or attentional modulation (Woods et al. 2009; Dick et al. 2017).

## Supplementary Material

Supplementary Data
