# Supplementary material for: Is Human Auditory Cortex Organization Compatible With the Monkey Model? Contrary Evidence From Ultra-High-Field Functional and Structural MRI
Source: Cereb Cortex. 2018 Oct 24;29(1):410–28. doi: 10.1093/cercor/bhy267 (PMC6294415; doi:10.1093/cercor/bhy267)
Supplement: Supplementary Data [file bhy267_supplementary_material_r3_v2.docx]

# Supplementary Figures and Tables

| Pattern | Pattern | Frequency | Examples | Figures |
| --- | --- | --- | --- | --- |
| A | Single low-frequency region on HG flanked by separate single high-frequency regions anteriorly and posteriorly | 8/24 | 6L, ***9L***, 10L, 12L, 6R, 7R, 8R, 9R | 4A,  S2A |
|  | Anterior high-frequency region is subdivided | 9/24 | 1L, ***3L***, 4L, 5L, 7L, 8L, 4R, 5R, 11R |  |
|  | Posterior high-frequency region is subdivided | 11/24 | 5L, 7L, 10L, ***11L***, 1R, 2R, 3R, 5R, 10R, 11R, 12R |  |
|  | Low-frequency region is subdivided | 5/24 | 1L, 2L, 1R, 2R, 10R |  |
|  | Anterior and posterior high-frequency regions are connected across the medial end of HG | 2/24 | ***5L***, 4R |  |
| B | Region of high frequency selectivity is elongated along the long axis of HG | 18/24 | 2L, ***3L***, 4L, ***5L***, 6L, 7L, 8L, ***9L***, 10L, 1R, 2R, 4R, 5R, 6R, 7R, 8R, 9R, 12R | 4C,  S2B |
|  | High-selectivity region is either not elongated or not elongated along HG | 6/24 | 1L, ***11L***, 12L, 3R, 10R, 11R |  |
| C | Region of high myelination (R1) is elongated along the long axis of HG | 17/24 | 1L, 2L, ***3L***, 4L, ***5L***, 6L, 7L, ***9L***, ***11L***, 12L, 2R, 4R, 5R, 6R, 8R, 9R, 12R | 4D,  S2C |
|  | High-myelin region is either not elongated or not elongated along HG | 7/24 | 8L, 10L, 1R, 3R, 7R,10R,11R |  |
| D | High-myelin region is restricted to HG | 11/24 | ***3L***, 8L, ***11L***, 12L, 2R, 3R, 4R, 8R, 9R, 11R, 12R |  |
|  | High-myelin region extends beyond the medial end of HG onto the insula and parietal lobe | 8/24 | 2L, 4L, ***5L***, 6L, 7L, ***9L***, 6R, 7R |  |
|  | High myelination is also present in a region posterior to HG (either connected to or separate from the region on HG) | 7/24 | 1L, 6L, 10L, 1R, 5R, 6R, 10R |  |
| E | High-myelin ROI shifted towards medial end of HG compared to high-selectivity ROI | 20/24 | 1L, 2L, ***3L***, 4L, ***5L***, 6L, 7L, 8L, ***9L***, 10L,12L, 1R, 2R, 3R, 4R, 5R, 6R, 7R, 8R, 9R | 4E,  S2D |
|  | High-myelin ROI not shifted relative to high-selectivity ROI | 4/24 | ***11L***, 10R, 11R, 12R |  |
| F | Single HG | 10/24 | 1L, 7L, 10L, ***11L***, 12L, 1R, 5R, 7R, 10R, 11LR |  |
|  | Partially duplicated HG (single medial stem) | 11/24 | 2L, ***3L***, 4L, ***5L***, 8L, ***9L***, 3R, 4R, 6R, 8R, 12R |  |
|  | Fully duplicated HG | 3/24 | 6L, 2R, 9R |  |

**Table S1**: Typical patterns of tonotopic, core and morphological configurations within individual hemispheres. The first column indicates the pattern category (A: tonotopy; B&C: orientations of high-selectivity and high-myelin regions in relation to Heschl’s gyrus (HG); D: medial extent of high-myelin region; E: relations between high-selectivity and high-myelin regions of interest (ROIs); F: HG morphology). The second column describes typical individual patterns, and the third and fourth columns give the number (out of 24) and labels of hemispheres showing the respective patterns. Bold labels indicate hemispheres depicted in the main manuscript, the other hemispheres are depicted in the supplementary figures (relevant figures are given in column five).

**
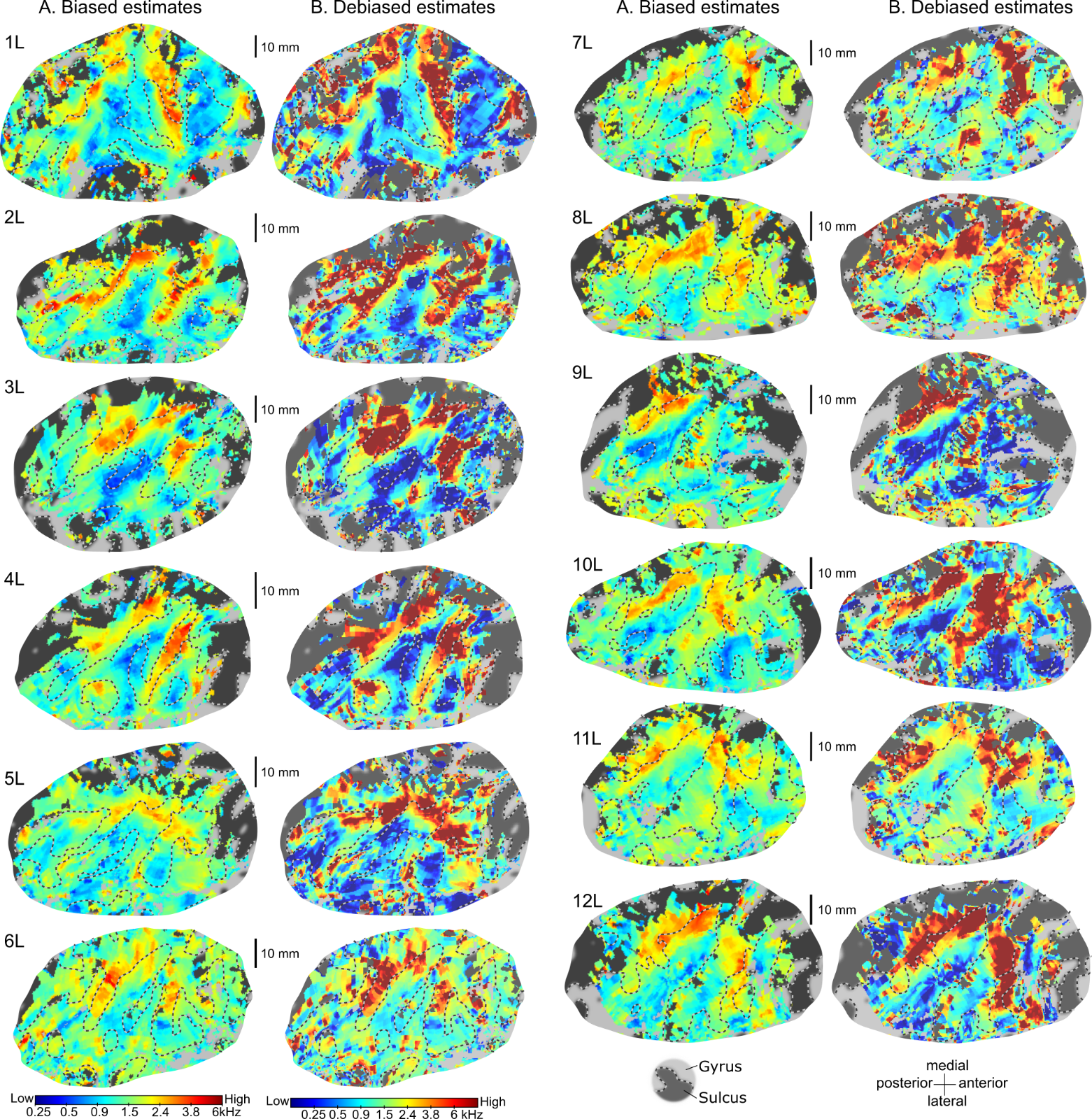
**

**Figure S1 (part 1)**: Comparison of biased (A) and unbiased (B) preferred-frequency estimates for all left hemispheres. The biased estimates are the tuning curve centroids, which are biased towards the middle of the stimulus frequency range. The debiasing procedure creates unbiased estimates across the entire stimulus range and beyond (see different color scales). Apart from the difference in scale, the patterns of the biased and unbiased estimates were highly similar.

**
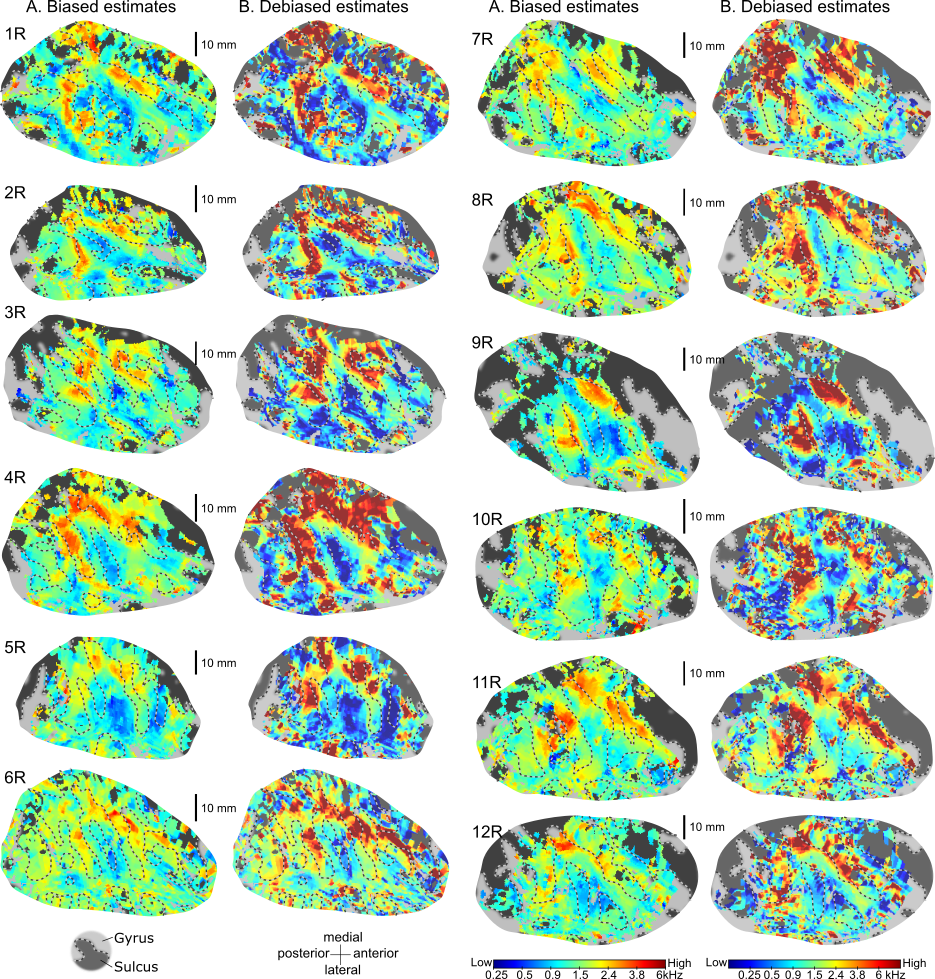
**

**Figure S1 (part 2)**: Same as Fig. S1 (part 1), but for the right hemispheres.


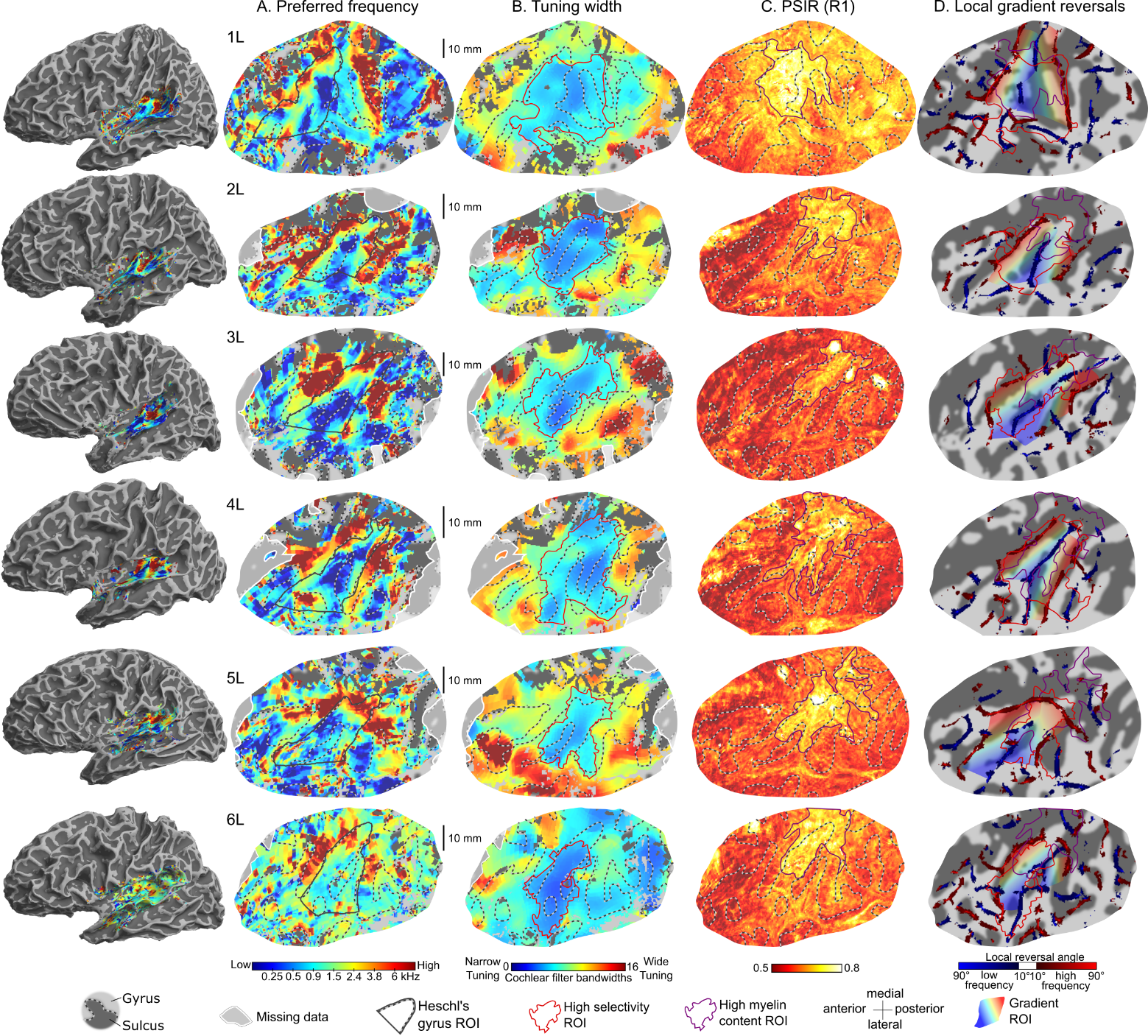


**Figure S2 (part 1)**: Individual functional and structural maps for left hemispheres 1L-6L, plotted in the same way as in Fig. 4, except that panel D now shows the gradient reversals, gradient ROIs and core ROIs all superimposed.

**
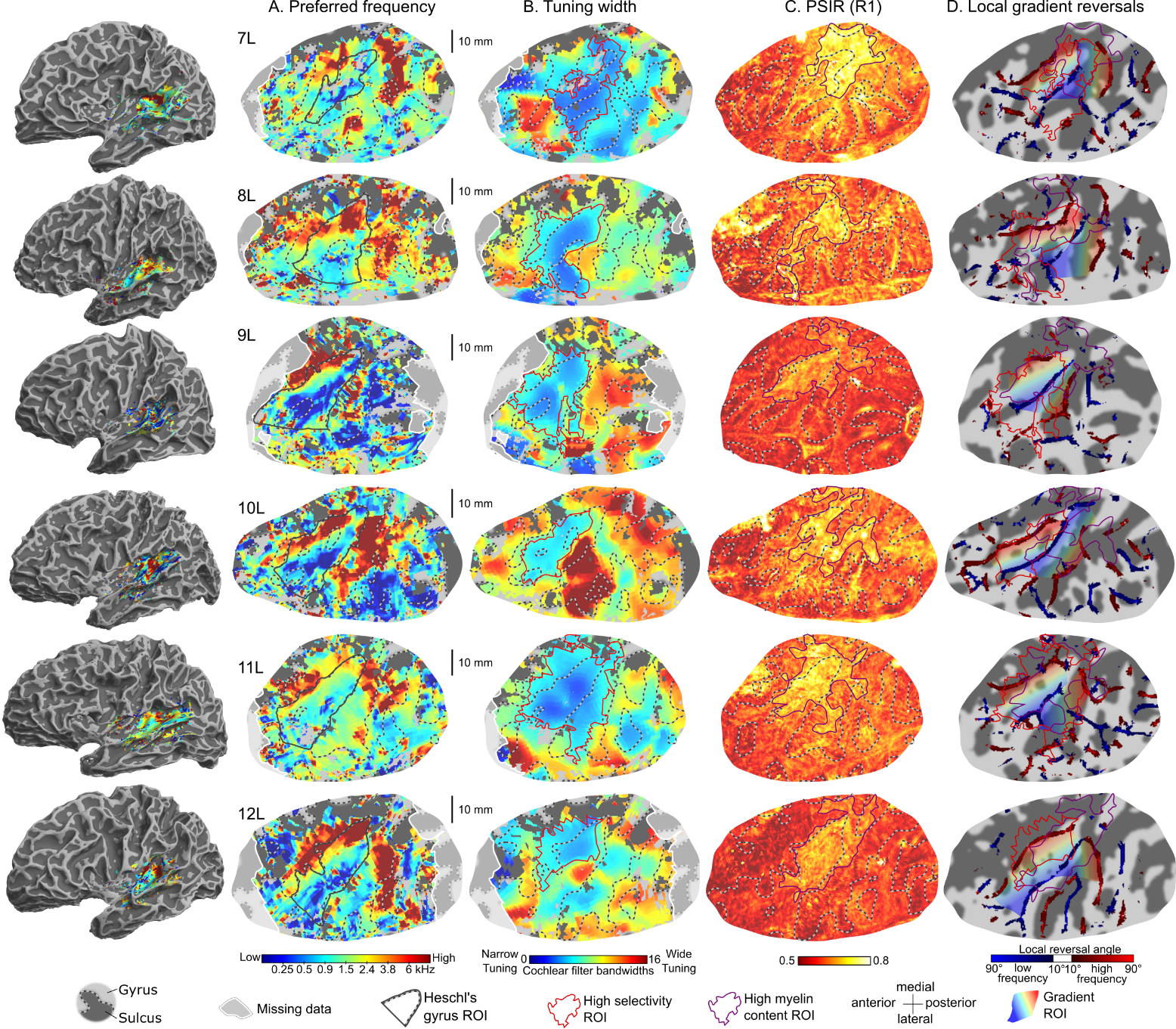
**

**Figure S2 (part 2)**: Same as Fig. S2 (part 1), but for left hemispheres 7L-12L.


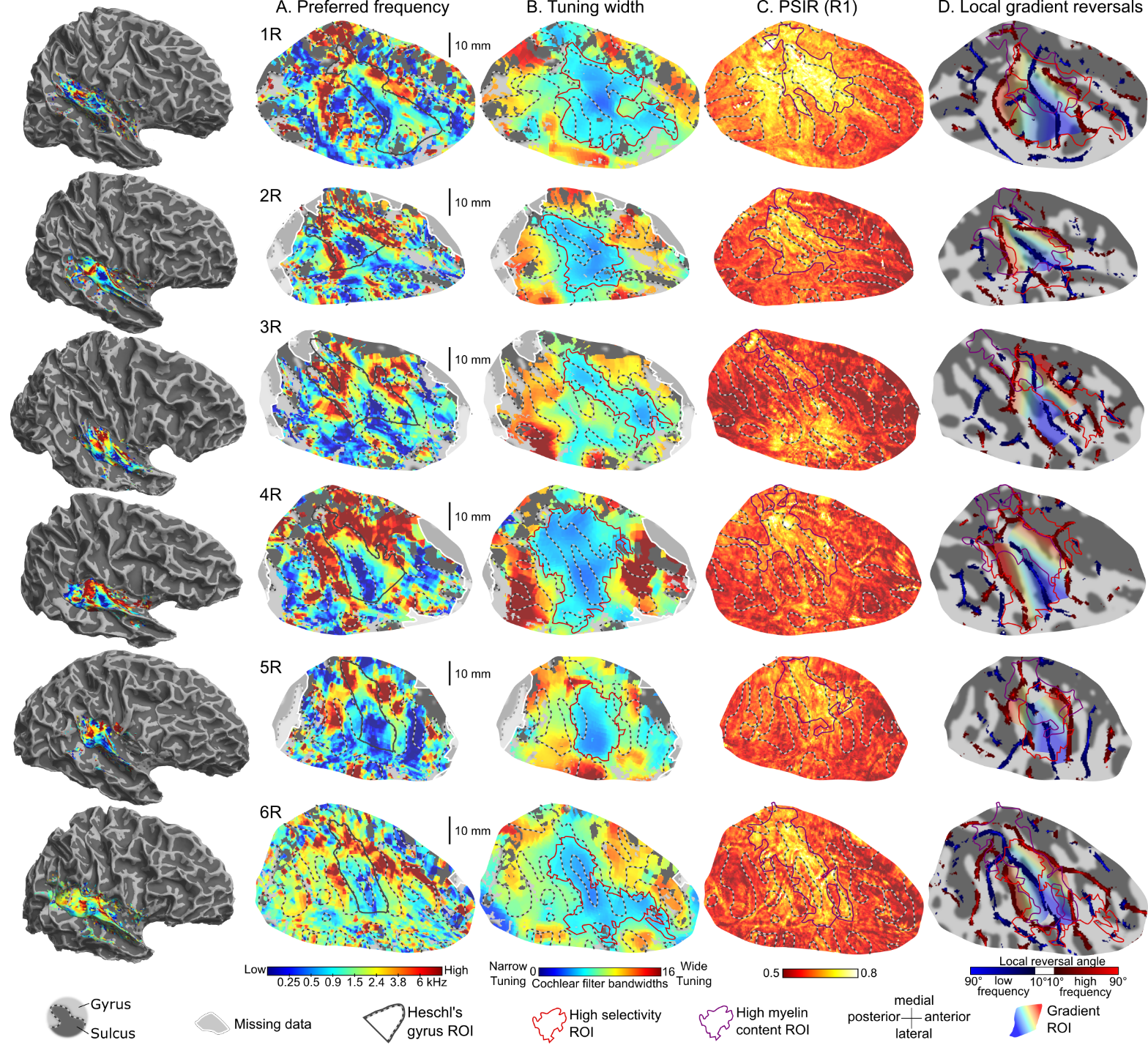


**Figure S2 (part 3)**: Same in Fig. S2 (part 1), but for right hemispheres 1R-6R.


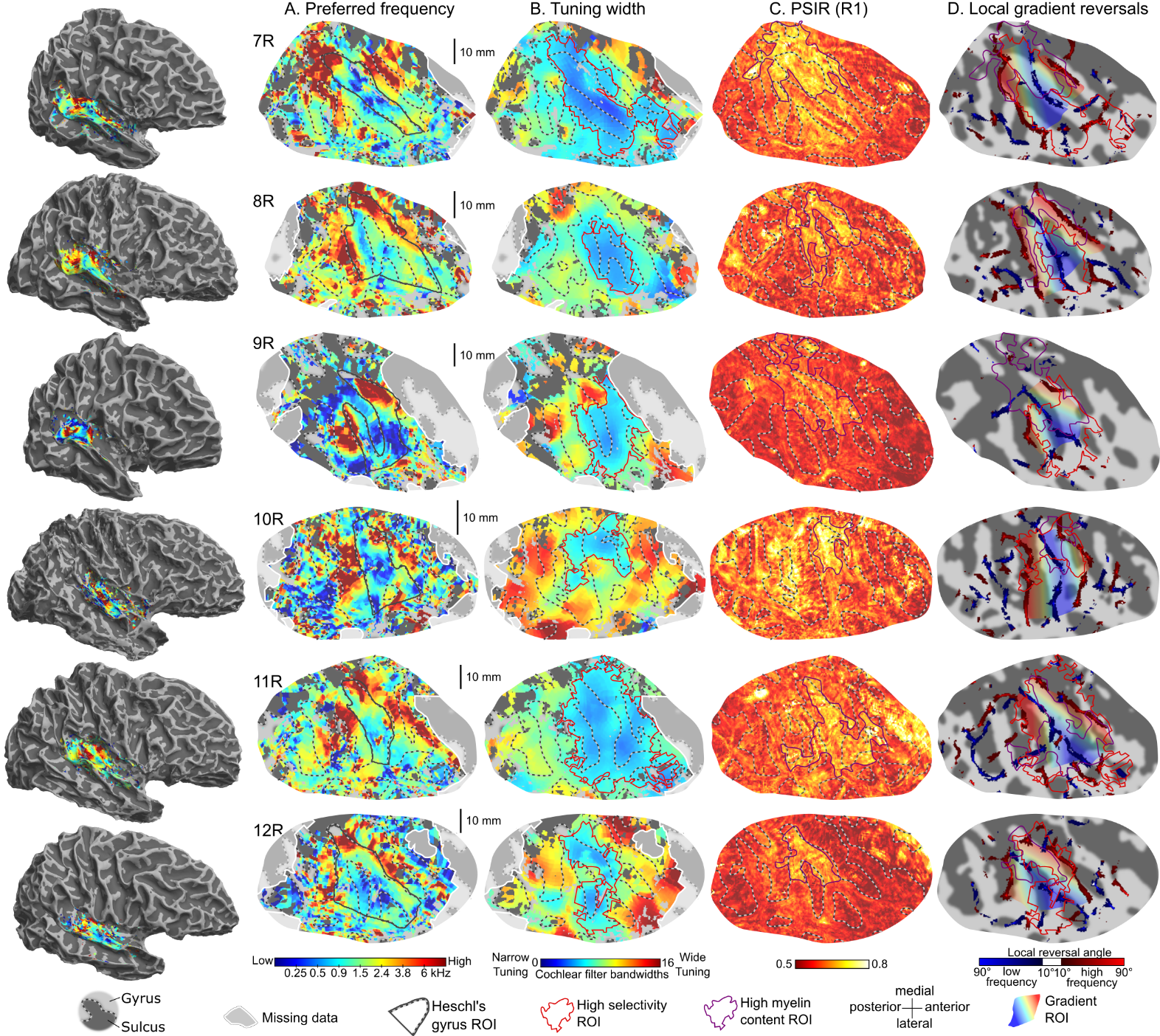


**Figure S2 (part 4)**: Same as Fig. S2 (part 1), but for right hemispheres 7R-12R.


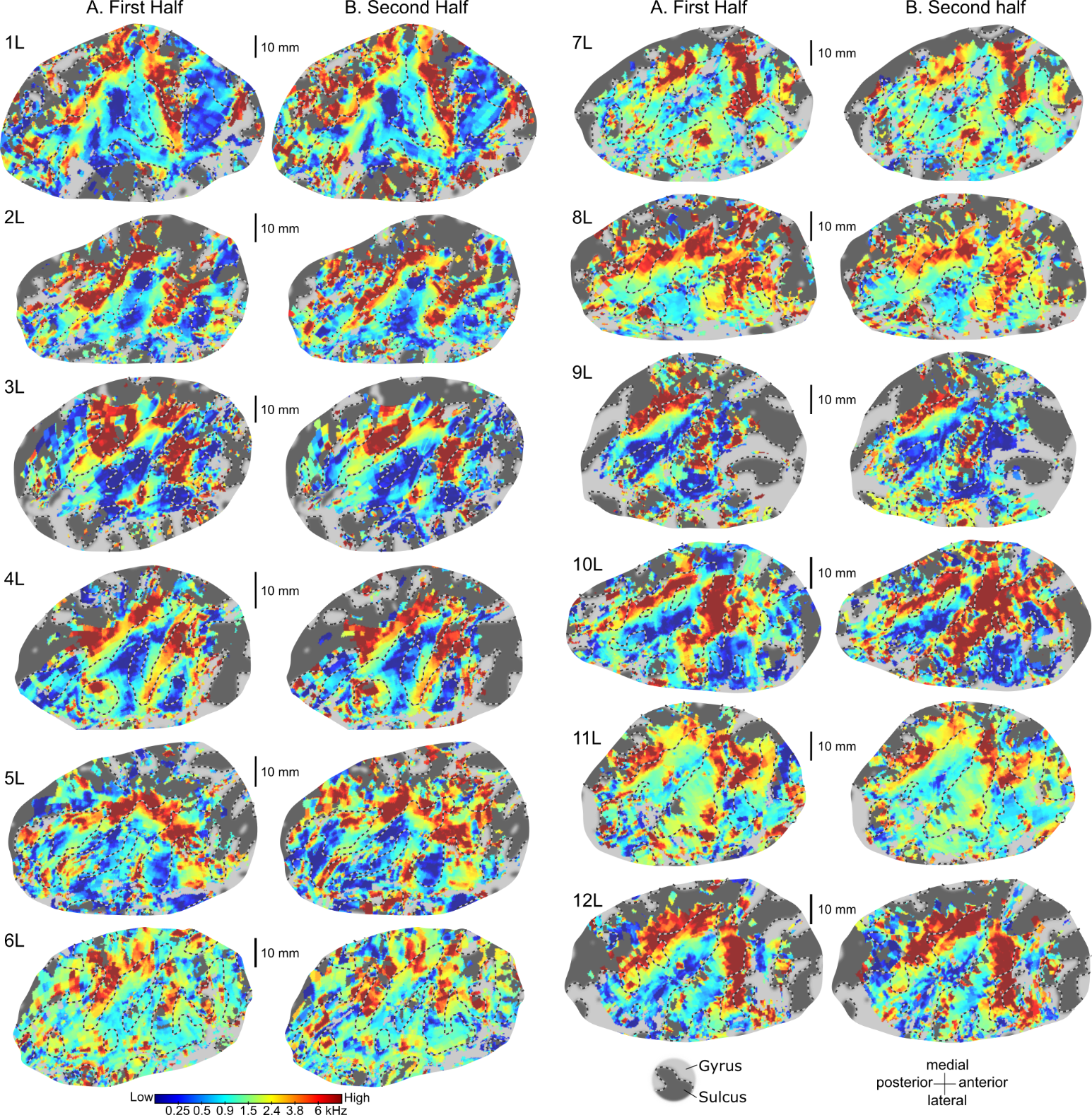


**Figure S3 (part 1)**: Split-half preferred-frequency maps for all left hemispheres. The split-half preferred-frequency estimates were calculated in the same way as for the individual maps in Figs 4A and S2A, but separately for the first (A) and second halves (B) of each individual’s dataset. The split-half maps are similar for all hemispheres.


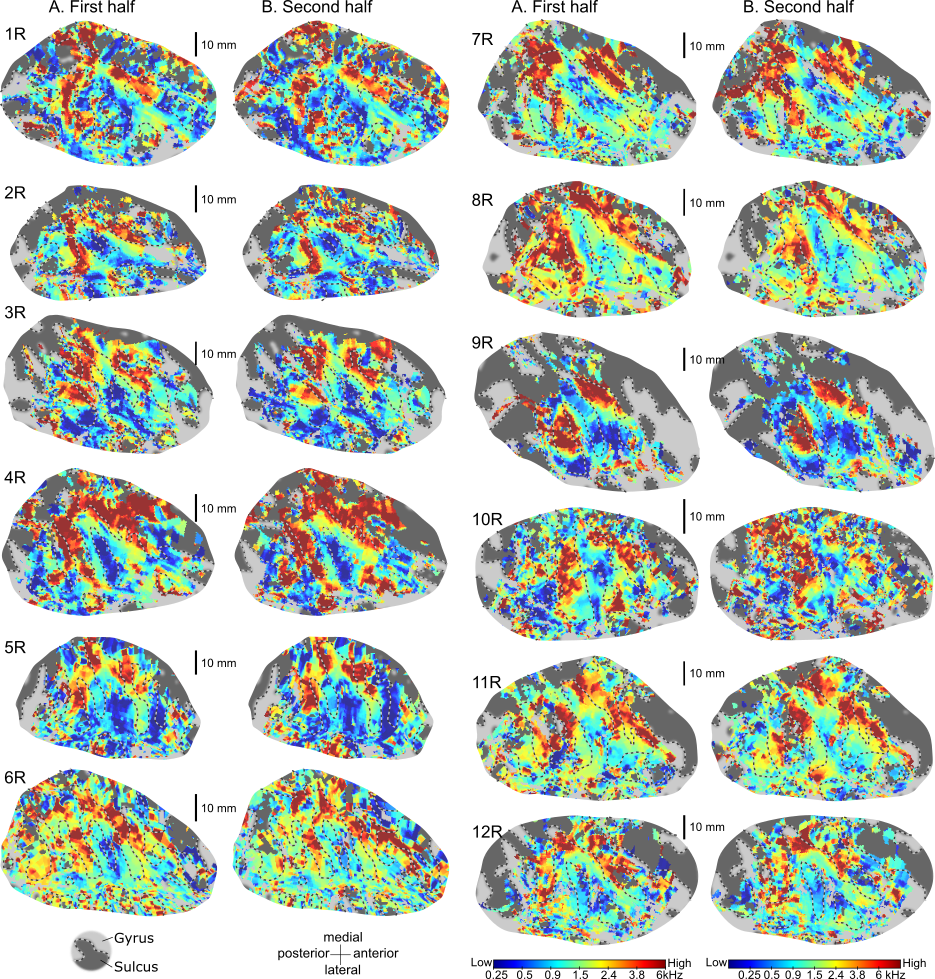


**Figure S3 (part 2)**: Same as Fig. S3 (part 1), but for the right hemispheres.

**
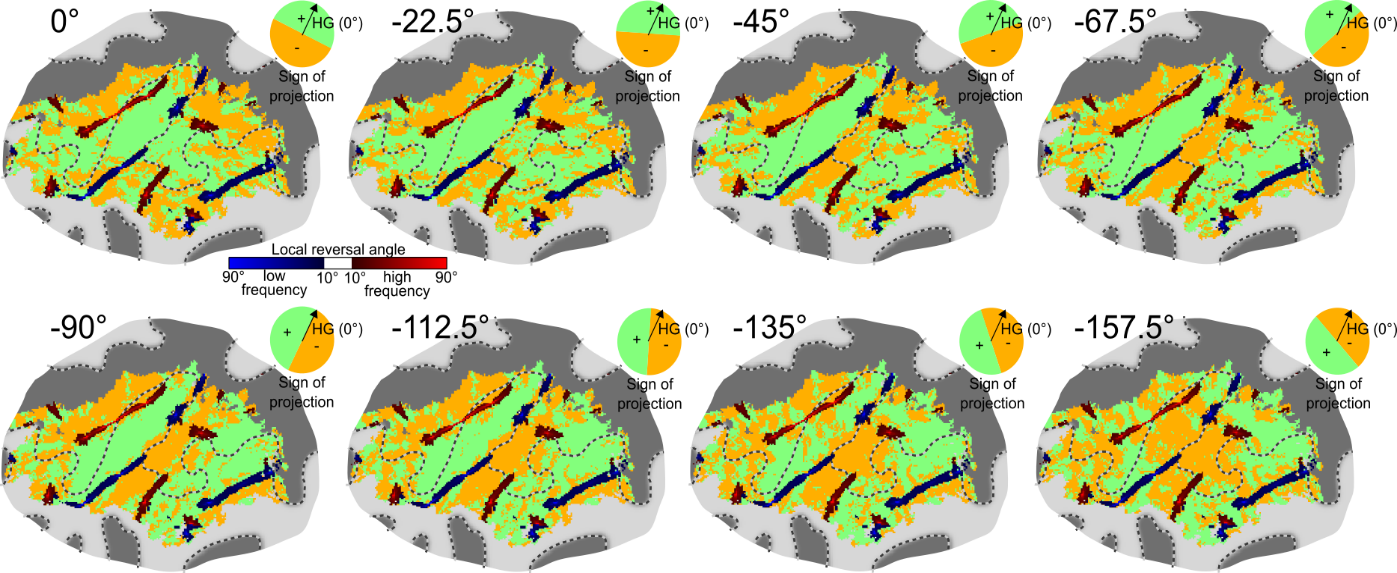
**

**Figure S4**: Group average gradient reversals, and gradient sign maps along 8 different directions from -90° to +67.5° relative to the direction of long axis of HG. Both the reversal and gradient sign maps were derived from the group-average gradient directions shown in Fig. 3B. When pixelwise group-average gradients were projected within 45° of the direction perpendicular to the long axis of HG (-45° to -112.5°), a pattern of mediolaterally extended strips of alternating gradient signs was observed, with borders between neighboring strips coinciding with gradient reversals. When projected onto other directions, the pattern and correspondence between gradient reversals and gradient sign borders breaks down.

**
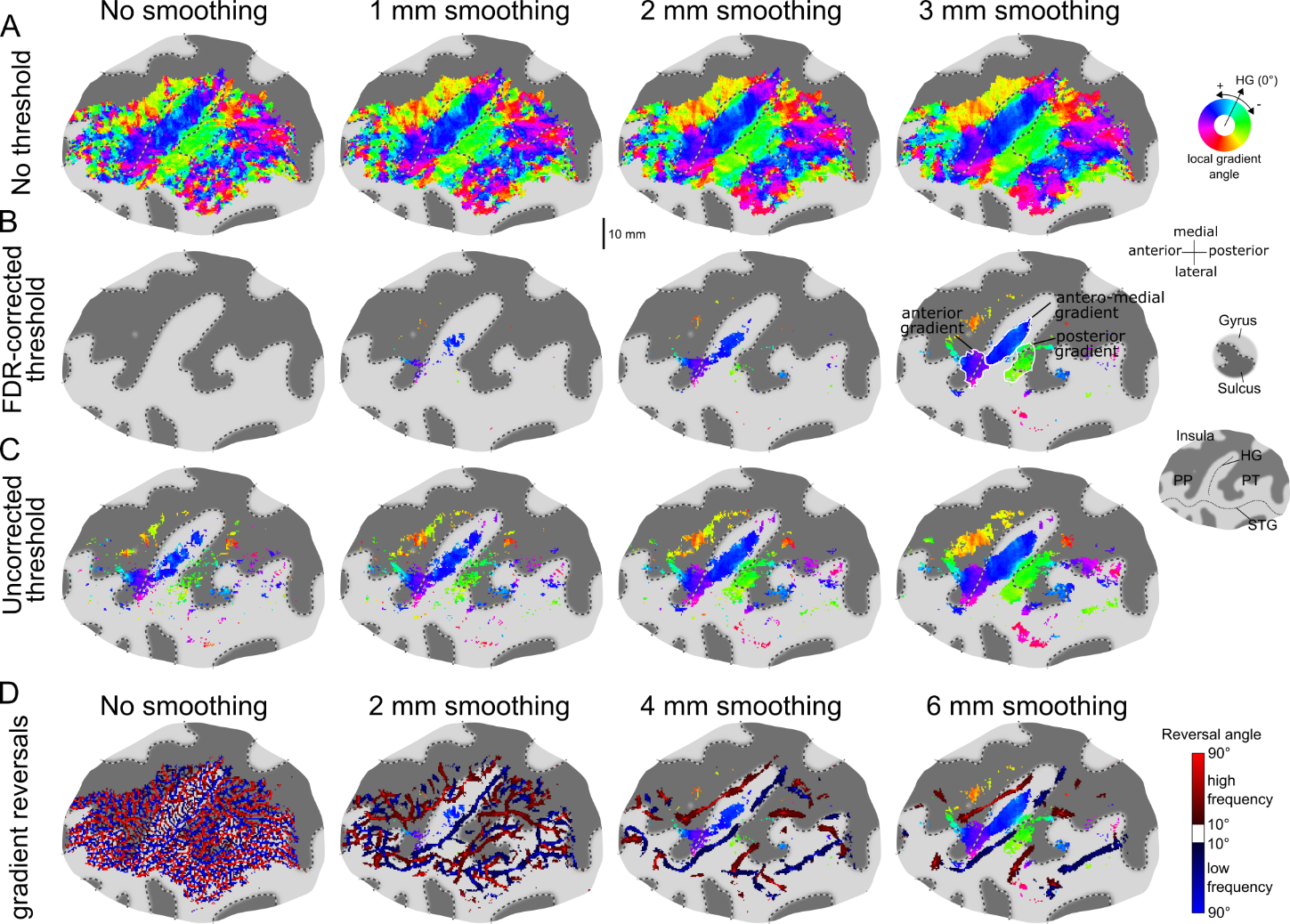
**

**Figure S5**: Effect of smoothing on group-average local tonotopic gradient directions and gradient reversals. A. Group-average local gradient directions (as in Fig. 3B) calculated for different two-dimensional (2D) Gaussian smoothing kernels [given in millimeters full width at half maximum (FWHM); the gradient directions in Fig. 3B were calculated without smoothing, as in the leftmost panel]. Gradient directions were similar across all levels of smoothing. B. Same as in A, but only showing gradient directions that were significantly consistent across hemispheres at p<0.05 (Hotelling T^2^), corrected for false discovery rate (FDR; see main text). Without any smoothing (leftmost panel), the corrected significance level was not reached at any pixel. C. Same as in B, but for an uncorrected significance level of *p* < 0.05. Significantly consistent gradient directions occurred within the same regions at all smoothing levels. D. Gradient reversals derived from the group-average preferred-frequency map for different smoothing kernels. The rightmost panel replots the reversals from Fig. 3C (calculated with a 6-mm kernel). When smoothing was reduced, reversals tended to break up into a random pattern, but the low-frequency reversal on HG (between the inter-individually consistent anterior and posterior gradients on HG) was robust down to the 2-mm smoothing kernel.


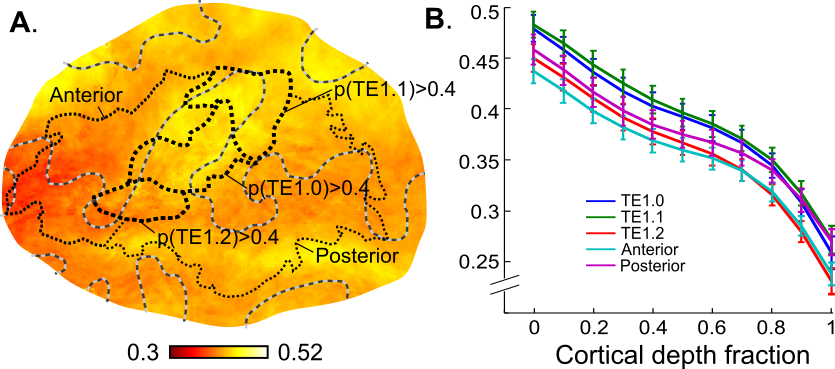


**Figure S6**: A. Group-average map of myelin content estimated with the magnetization transfer ratio (MTR). As in Fig. 3D&E, the thick black dotted lines show ROIs based on the cytoarchitectonically-defined areas TE1.0, TE1.1 and TE1.2 and the thin black dotted lines represent auditory-responsive ROIs anterior and posterior to these areas (referred to as “Anterior” and “Posterior”). B. ROI-average MTR, expressed as a function of cortical depth (0 = pial surface, 1 = gray/white matter boundary). Error bars represent the standard error of the mean across hemispheres.

**
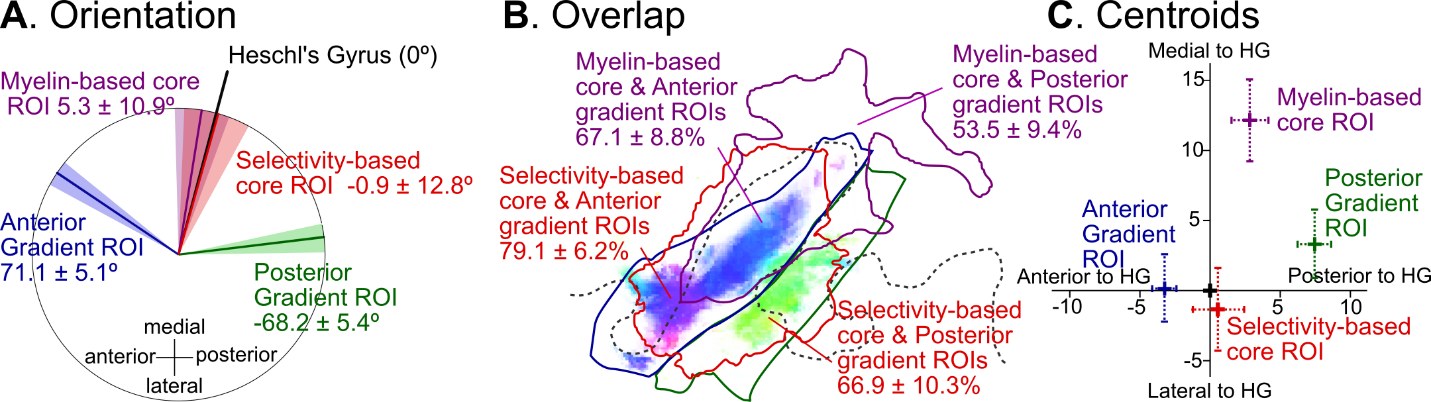
**

**Figure S7**: Orientations, overlap, and centroids of individual core and gradient ROIs, as in Fig. 5, but using a more inclusive threshold criterion for defining the core ROIs. In Fig. 5, the criterion was set midway between the average core marker values within area TE1.0 on the one hand, and the auditory responsive areas outside the conjunction of the three TE1 areas on the other. Here, it was instead set midway between the average marker values within the conjunction of the three TE1 areas and their complement (auditory-responsive regions outside these areas). The pattern of results is similar to that for the original criterion (compare with Fig. 5).

**
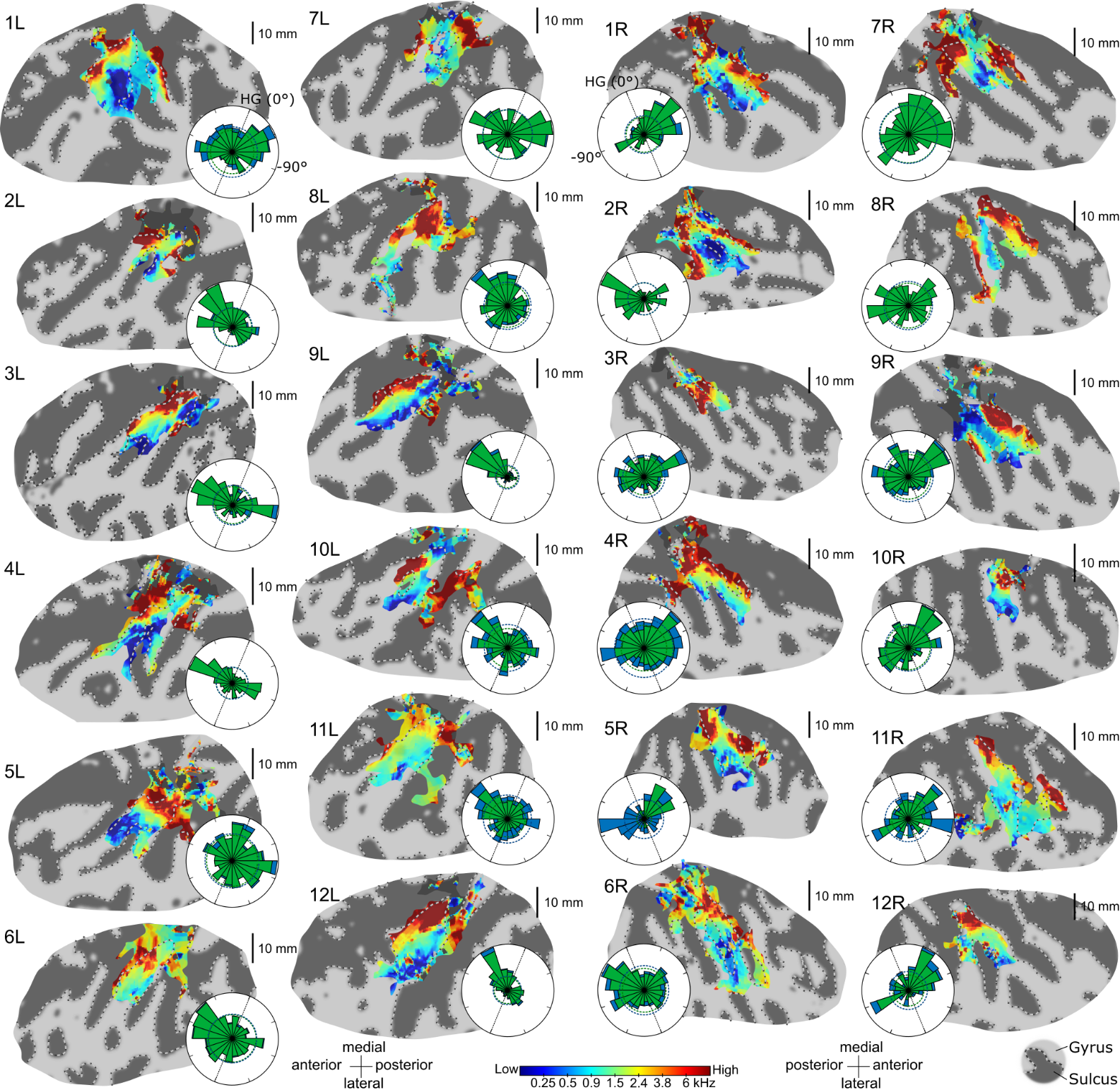
**

**Figure S8**: Individual preferred frequencies and local gradient directions within the high-myelin core ROI, as in Fig. 6A, shown for all hemispheres. In this case, the insets show gradient direction histograms for both the original (green), and the more inclusive (blue), definition of the high-myelin ROI (see caption of Fig. S7 for an explanation of the two definitions).

**
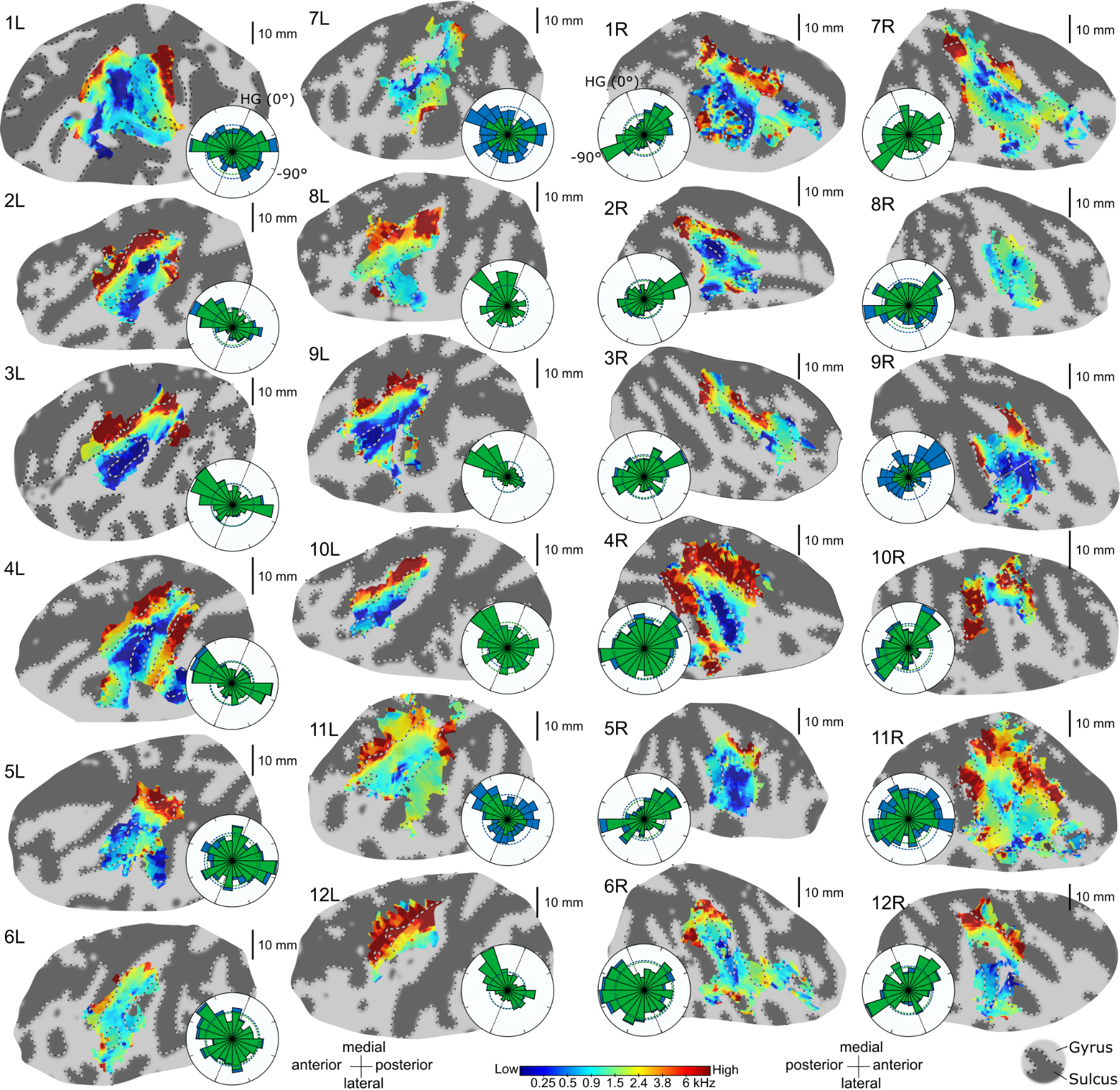
**

**Figure S8 (part 2)**: Same as Fig. S8 (part 1), but for the high-selectivity ROI.

**
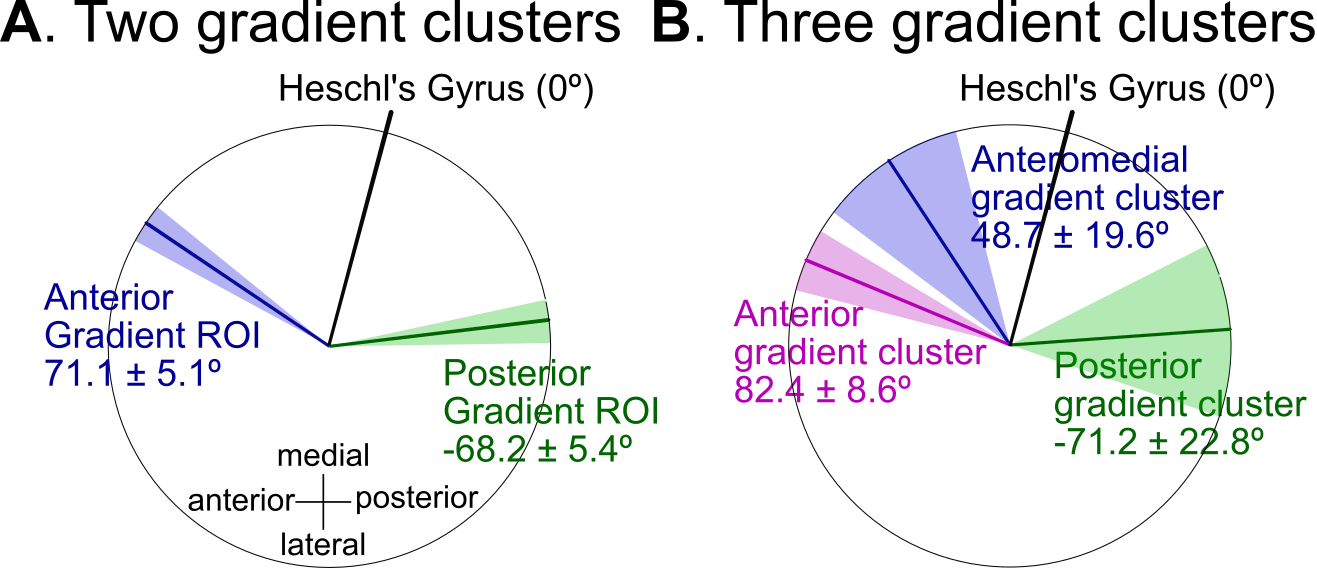
**

**Figure S9**: Average gradient orientations within two versus three gradient clusters. A. Gradient orientations averaged across the anterior and posterior gradient ROIs and across individual hemispheres (replotted from Fig 5A). B. Gradient orientations averaged across the posterior significantly consistent gradient cluster as well as the anteromedially and anteriorly pointing parts of the significantly consistent anterior cluster (see white outlines in the rightmost panel of Fig. S5B). The cluster gradient orientations (B) were calculated within each individual hemisphere separately and then averaged across hemispheres using the same procedure as for the ROI gradient orientations (A), except that the clusters were defined in the normalized coordinate spaces (whereas the gradient ROIs were defined in the spaces of the individual hemispheres using the automatically-detected gradient reversals). The average gradient direction in the posterior cluster was similar to that in the posterior gradient ROI. Note, however, the remarkable differences in the size of the 95% confidence intervals. The average gradient directions in the anteromedially and anteriorly pointing clusters were significantly different from each other, but formed a sharp angle. In contrast, each formed an obtuse angle with the average gradient direction in the posterior cluster. Both obtuse angles were approximately mirror-symmetric about the long axis of HG.
